# Insights into the ancestry evolution of the *Mycobacterium tuberculosis* complex from analysis of *Mycobacterium riyadhense*

**DOI:** 10.1093/nargab/lqab070

**Published:** 2021-08-11

**Authors:** Qingtian Guan, Musa Garbati, Sara Mfarrej, Talal AlMutairi, Thomas Laval, Albel Singh, Shamsudeen Fagbo, Alicia Smyth, John A Browne, Muhammad Amin urRahman, Alya Alruwaili, Anwar Hoosen, Conor J Meehan, Chie Nakajima, Yasuhiko Suzuki, Caroline Demangel, Apoorva Bhatt, Stephen V Gordon, Faisal AlAsmari, Arnab Pain

**Affiliations:** Pathogen Genomics Laboratory, BESE Division, King Abdullah University of Science and Technology (KAUST), Thuwal-Jeddah, 23955, Saudi Arabia; King Fahad Medical City (KFMC), Riyadh, 11525, Saudi Arabia; Pathogen Genomics Laboratory, BESE Division, King Abdullah University of Science and Technology (KAUST), Thuwal-Jeddah, 23955, Saudi Arabia; King Fahad Medical City (KFMC), Riyadh, 11525, Saudi Arabia; Immunobiology of Infection Unit, Institut Pasteur, INSERM U1221, Paris, France; Université Paris Diderot, Sorbonne Paris Cité, 75205 CEDEX 13, Paris, France; Institute of Microbiology and Infection, School of Biosciences, University of Birmingham, B15 2TT, Edgbaston, Birmingham, UK; One Health Unit, Executive Directorate for Surveillance and Response, Saudi Center for Disease Prevention and Control, 13352, Riyadh, Saudi Arabia; Department of Public Health, Nigerian Institute of Medical Research, P.M.B. 2013, Yaba, Lagos, Nigeria; UCD School of Veterinary Medicine, University College Dublin, Dublin, D04 W6F6, Ireland; UCD School of Agriculture and Food Science, University College Dublin, Dublin, D04 W6F6, Ireland; King Fahad Medical City (KFMC), Riyadh, 11525, Saudi Arabia; King Fahad Medical City (KFMC), Riyadh, 11525, Saudi Arabia; King Fahad Medical City (KFMC), Riyadh, 11525, Saudi Arabia; School of Chemistry and Biosciences, University of Bradford, Bradford, BD7 1AZ, UK; Global Institution for Collaborative Research and Education, Hokkaido University, Kita 20 Nishi 10, Kita-ku, Sapporo, Japan; Research Center for Zoonosis Control, Hokkaido University, Kita 20 Nishi 10, Kita-ku, Sapporo, Japan; Global Institution for Collaborative Research and Education, Hokkaido University, Kita 20 Nishi 10, Kita-ku, Sapporo, Japan; Research Center for Zoonosis Control, Hokkaido University, Kita 20 Nishi 10, Kita-ku, Sapporo, Japan; Immunobiology of Infection Unit, Institut Pasteur, INSERM U1221, Paris, France; Institute of Microbiology and Infection, School of Biosciences, University of Birmingham, B15 2TT, Edgbaston, Birmingham, UK; UCD School of Veterinary Medicine, University College Dublin, Dublin, D04 W6F6, Ireland; Global Institution for Collaborative Research and Education, Hokkaido University, Kita 20 Nishi 10, Kita-ku, Sapporo, Japan; King Fahad Medical City (KFMC), Riyadh, 11525, Saudi Arabia; Pathogen Genomics Laboratory, BESE Division, King Abdullah University of Science and Technology (KAUST), Thuwal-Jeddah, 23955, Saudi Arabia; Global Institution for Collaborative Research and Education, Hokkaido University, Kita 20 Nishi 10, Kita-ku, Sapporo, Japan

## Abstract

Current evolutionary scenarios posit the emergence of *Mycobacterium tuberculosis* from an environmental saprophyte through a cumulative process of genome adaptation. *Mycobacterium riyadhense*, a related bacillus, is being increasingly isolated from human clinical cases with tuberculosis-like symptoms in various parts of the world. To elucidate the evolutionary relationship between *M. riyadhense* and other mycobacterial species, including members of the *M. tuberculosis* complex (MTBC), eight clinical isolates of *M. riyadhense* were sequenced and analyzed. We show, among other features, that *M. riyadhense* shares a large number of conserved orthologs with *M. tuberculosis* and shows the expansion of toxin/antitoxin pairs, PE/PPE family proteins compared with other non-tuberculous mycobacteria. We observed *M. riyadhense* lacks *wecE* gene which may result in the absence of lipooligosaccharides (LOS) IV. Comparative transcriptomic analysis of infected macrophages reveals genes encoding inducers of Type I IFN responses, such as cytosolic DNA sensors, were relatively less expressed by macrophages infected with *M. riyadhense* or *M. kansasii*, compared to BCG or *M. tuberculosis*. Overall, our work sheds new light on the evolution of *M. riyadhense*, its relationship to the MTBC, and its potential as a system for the study of mycobacterial virulence and pathogenesis.

## INTRODUCTION

The *Mycobacterium tuberculosis* complex (MTBC) is a group of genetically related pathogens that cause tuberculosis (TB) in mammalian species. The hallmark member, *Mycobacterium tuberculosis*, is the single most deadly bacterial pathogen, causing over 1.4 million deaths globally in 2019. Current evolutionary scenarios posit the evolution of the MTBC from an environmental saprophyte through a cumulative process of genome adaptation. Such scenarios envisage intermediate mycobacterial species with increasing pathogenic potential for humans, the vestiges of which should be present in extant mycobacterial species. Comparative genomic analyses between the MTBC members and opportunistic mycobacterial pathogens may therefore reveal the key evolutionary steps involved in the emergence of the MTBC, as well as illuminating virulence mechanisms across mycobacterial pathogens as a whole.

Non-tuberculous mycobacteria (NTMs) are naturally occurring environmental bacteria commonly found in water and soil. A wide range of animal and environmental sources (aquaria, swimming pools) act as reservoirs for NTMs, and several human disease outbreaks caused by exposure to environmental NTMs have been described (1). Little is known about the natural reservoir of *M. riyadhense* except for two studies that describe *M. riyadhense* being isolated from water (2) and soil (3). Since its discovery in 2009, *M. riyadhense* has been revealed to be a clinically important pathogen, able to cause disease in both immunocompromised and immunocompetent individuals (4–6), The clinical and radiologic characteristics of pulmonary infection caused by *M. riyadhense* are indistinguishable from those caused by *M. tuberculosis*, the most important human pathogen of the MTBC (4,7).

Similar to *M. tuberculosis*, *M. riyadhense* grows at 37°C and requires 2–3 weeks (8) to form visible colonies on agar media. No evidence of human-to-human transmission yet reported. Infections with *M. riyadhense* have been reported in Asia and Europe in countries including Bahrain, South Korea, France, Italy and Germany (7,9,10), although most of the recent cases originated in patients from Saudi Arabia. Indeed, the very first case of acute *M. riyadhense* infection was initially misdiagnosed as a case of *M. tuberculosis* infection in a Saudi hospital using commercially available diagnostic tests (4).

It is postulated that *M. tuberculosis* evolved from a free-living environmental ancestor into an obligate pathogen (11). In a recent publication, Sapriel and Brosch (12) showed the close phylogenetic relationship of *M. riyadhense* to the MTBC, suggesting that it forms part of an MTB-associated phylotype (MTBAP) with *Mycobacterium decipiens*, *Mycobacterium lacus* and *Mycobacterium shinjukuense*. The MTBAP have only been isolated from human clinical samples, and share specific traits that differentiate them from environmental NTMs; they hence likely serve as evolutionary intermediates between the environmental mycobacteria and the pathogenic members of the MTBC.

*Mycobacterium* species have a complex cell wall that contains unusual lipids, such as lipo-oligosaccharides (LOSs), and functions as a permeability barrier. Studies have shown that the LOS-deficient rough variant of *M. kansasii* persists longer in infected mice compared with the LOS-producing smooth strain (13). Defects in higher order LOS biosynthesis have also been correlated with smooth-wrinkled phenotype (14). Modern *M. tuberculosis* complex strains are more hydrophobic compared to *M. canettii* that contains abundant LOSs. This change of polarity/hydrophobicity is thought to increase their capability for aerosol transmission, affecting their virulence and pathogenicity (15). It is thus speculated that the loss of LOSs was an important step in the emergence of pathogenic mycobacteria.

Mycobacteria contain specialized Type VII secretion (T7S) systems (ESX1-5) for transportation of proteins (harbouring WXG motifs) through the hydrophobic cell wall (16). ESX systems mediate a broad range of functions in mycobacteria. ESX-1 in *M. tuberculosis* that is linked with growth within macrophages (17), cytosolic translocation (18) and antigen presentation (19). It has also been shown that ESX-1 plays a role in DNA transfer in *M. smegmatis* (20). ESX-3 contributes to iron acquisition in mycobacteria, as well as cell survival (21) and virulence (22) in pathogenetic mycobacteria. ESX-5 plays a major role in the capsule integrity through its substrate PPE10 (23). The functions of ESX-2 and ESX-4 remain to be fully elucidated.

To study the distribution of the virulence factors in *M. riyadhense* and other mycobacteria from an evolutionary context, we investigated the presence and absence of several virulence factors using comparative genomics approaches. Pathogenic *Mycobacterium* species have developed strategies to avoid or modulate the host immune response through virulence factors that include lipids, secretion systems and their secreted effectors, and cell surface molecules. For example, various PE/PPE family proteins, named after the conserved proline-glutamate (PE) or proline-proline-glutamate (PPE) motifs at their N-terminus, have been shown to be expressed by *M. tuberculosis* upon infection of macrophages and play critical roles in virulence, antigenic diversity and modulation of the host immune response (24). Another group of virulence factors is the mammalian cell entry (*mce*) proteins, which play an important role in the host cell signalling modulation as well as their primary role as lipid/sterol transporters (25).

Toxin/antitoxin (T/A) systems were first found on plasmids or plasmid-derived chromosomal loci where they were implicated in plasmid maintenance in bacterial populations (26). When compared to other mycobacteria, the MTBC members are remarkable for the extensive repertoire of T/A systems. A hallmark of *M. tuberculosis* infection is the ability to survive long-term in host granulomas and develop a latent stage of infection. The molecular mechanisms and cellular components that are involved in the persistence of *M. tuberculosis* are still poorly understood, but several T/A systems have been implicated in the pathogenicity of *M. tuberculosis* (27).

To investigate the MTBC evolutionary history, we report the characterization of eight clinical isolates of *M. riyadhense*, compare their genomes to members of the MTBC, and provide insights into the speciation of the MTBC from MTBAP and environmental bacteria. Due to their suggested role in virulence, we examined the LOS profiles of both rough and smooth variants of *M. riyadhense*, comparing them to those of other related mycobacteria. We furthermore analyzed the comparative transcriptional response of immunity-related host genes in a murine macrophage infection model after infection with *M. riyadhense*, *M. kansasii*, *M. bovis* BCG or *M. tuberculosis*.

To facilitate ease of identification of *M. riyadhense*, we also built on our genomic analyses to develop a simple PCR-based diagnostic test for the rapid and accurate identification of *M. riyadhense* so as to minimize the risk of misdiagnosis in a clinical setting.

Our analyses provide a comprehensive description of the hallmarks of *M. riyadhense* that make it one of the closest known environmental relatives of the MTBC, and that can serve to illuminate studies into the evolution and pathogenesis of the MTBC.

## MATERIALS AND METHODS

### Ethics statement

The research protocol was approved by the Institutional Review Board of King Fahad Medical City (Riyadh, Saudi Arabia; #16–345) and the Institutional Biosafety and Bioethics Committee of King Abdullah University of Science and Technology (Jeddah, Saudi Arabia; #18IBEC23). All adult subjects provided informed and written consent. A parent or guardian of any child participant provided informed consent on their behalf.

### Culturing, DNA isolation and sequencing of bacteria

Eight *M. riyadhense* strains were collected in Riyadh, Saudi Arabia, between June 2011 and March 2016 from patients with a positive culture for *M. riyadhense* isolated from the microbiology laboratory at the King Fahad Medical City (KFMC) in Riyadh, Saudi Arabia. The *M. riyadhense* strains were grown on Lowenstein Jensen (LJ) slants at 37°C for 2 weeks, DNA was extracted using a phenol-chloroform protocol, and the quality was measured by Qubit. Twenty micrograms of high-molecular-weight DNA from the eight *M. riyadhense* strains were sequenced using a PacBio RSII sequencer (Pacific Biosciences, Menlo Park, U.S.A.) with a 10 kb library. A NEBNext Ultra II DNA library preparation kit (New England BioLabs, Massachusetts, U.S.A.) was used to prepare libraries according to the manufacturer’s instructions, and sequences from each library were generated for all *M. riyadhense* strains using the Illumina HiSeq 4000 platform (Illumina, San Diego, U.S.A.).

### Genome assembly and annotation

The Illumina short reads were trimmed, and low-quality reads were removed by Trimmomatic (28). We did *de novo* assemblies of the eight consensus genomes of each *M. riyadhense* strain with the PacBio long reads using the Canu assembler (29). After assembly, the draft genomes were subsequently corrected with short Illumina reads using the Pilon (30) software. The circularity of assemblies was checked by Gepard (31), and assemblies were annotated by Prokka (32). A circular map of the chromosome was compared with that of *M. tuberculosis* H37Rv and visualized with BRIG (33). For the comparative genomics analysis, the genome of the *M. riyadhense* MR226 strain was used as a high-quality representative reference in this study. The overall study design flowchart is illustrated in Supplementary Figure S1.

### Phylogenetic, comparative genomics and *M. tuberculosis* T/A orthologs analysis

We obtained draft genomes from 146 *Mycobacterium* species from the National Center for Biotechnology Information (NCBI) database and independently annotated by Prokka (32) (except for the *M. tuberculosis* H37Rv, *M. marinum* M, *M. kansasii* ATCC12478 and the genomes of MTBC members) for phylogenetic analysis and to identify the members of the genes belonging to the *mce* and the *pe/ppe* families. Accession numbers for these genomes can be found in Supplementary Table S1.

Protein homology of *M. tuberculosis* H37Rv *esx1-5* loci were detected by Proteinortho (34) across all the mycobacterial genomes. The presence of *esx* loci was defined such that at least three orthologs of genes present in *M. tuberculosis esx* loci were in close proximity to one another in the genome. The PhyoPhlAn2 (35) pipeline was used construct the phylogeny of the 147 (including the *M. riyadhe*nse strain MR226) genomes and *Nocardia abscessus* was used as the out-group. PhyloPhlAn2 was used to generate the phylogenetic tree by concatenating the informative amino-acid positions from 400 marker genes and aligned separately by MUSCLE (36) and reconstructed into trees using FastTree (37) and RaxML (38).

A focused OrthoMCL (39) comparison was performed between (i) *M. riyadhense*, *M. marinum*, *M. kansasii*, *M. szulgai* and *M. tuberculosis* and (ii) *M. riyadhense* and five species from the MTBC, namely, *M. tuberculosis*, *M. bovis* BCG, *M. canettii*, *M. mungi* and *M. africanum*.

To study the presence of *M. tuberculosis* orthologs in the genus *Mycobacterium*, 158 T/A proteins belonging to the VapBC, RelEF, HigBA, MazEF, ParDE and UCAT families that have been described previously (40) were retrieved from the *M. tuberculosis* H37Rv assembly. The *M. tuberculosis* T/A orthologs from all of the 147 species were calculated by Proteinortho (34) (Supplementary Material online).

### SNP calling and phylogeny based on SNPs

The corrected Illumina reads were mapped using BWA (41) onto the *M. riyadhense* MR226 genome assembly. Picard tool (42) was used to clean SAM files, fix mate-pair information and mark duplicates. SNPs were called for two iterations and filtered according to the Genome Analysis Toolkit (GATK) (43) pipeline recommendations. Phylogeny was generated by RaxML (44) with the TVM model.

### Infection of the RAW 246.7 cell line

The murine macrophage RAW264.7 cell line was obtained from the American Type Culture Collection (ATCC, Manassas, U.S.A.) and cultured in Dulbecco’s modified Eagle’s medium (DMEM) (ThermoFisher Scientific, Waltham, U.S.A.) supplemented with 10% FCS, streptomycin and penicillin. *Mycobacterium riyadhense* MR226, *M. kansasii* (subtype I), *M. bovis* BCG Denmark and *M. tuberculosis* H37Rv strains were grown in Middlebrook 7H9 liquid medium after single-colony isolation from LJ slants or 7H10 agar. 7H9 was supplemented with 10% albumin, dextrose and catalase (ADC), while 7H10 was supplemented with oleic acid, albumin, dextrose and catalase (OADC) in addition to 0.2% glycerol. The infection experiment was carried out according to the experimental conditions described previously (45). The supernatant was removed after 3 h, and the infected cells were washed with PBS to remove extracellular bacteria. Subsequently, the cells were incubated in fresh DMEM with 10% FCS for 24 and 48 h. All of the experiments were carried out in three different independent batches and used as triplicates in the study. For harvesting, 400 μl of TRIzol (ThermoFisher Scientific, Waltham, U.S.A.) was added to the wells at each time point, and the adherent cells were scraped out and stored at -80°C for RNA extraction. Each bacterial infection was performed in triplicate, in addition to the non-infected controls.

### RNA preparation and RNAseq analysis

RNA was isolated from the samples using the Direct-zol™ RNA Miniprep kit (Zymo Research, Irvine, U.S.A.) according to the manufacturer’s instructions. An Agilent RNA 6000 Nano kit was used to check the quality and quantity of the total RNA. The RNA libraries were prepared by following the Illumina TruSeq RNA Sample Preparation Kit v2 protocol.

The Illumina short reads were trimmed, and low-quality reads were removed by Trimmomatic (28). The RNAseq reads were mapped to GRCm38 reference genome with HISAT2 (46).The number of reads that mapped to each annotated gene was counted using the HTSeq (47) with ‘union’ overlap resolution mode. Data were normalized and the DE genes were identified using DESeq2 package (48). A fold-change in expression ≥2 with an adjusted *P*-value (padj) < 0.01 was considered as significant, and genes fulfilling these criteria were considered to be differentially expressed (DE) upregulated genes. The functional annotation of the DE genes were then analysed using the Metascape (49).

### Thin-layer chromatography analysis of lipooligosaccharides in *M. riyadhense*, *M. kansasii* and *M. marinum*

For TLC analysis, mycobacterial strains were grown at 30°C (*M. marinum* M) or 37°C (*M. smegmatis* MC-1551, *M. kansasii* subtype I, *M. riyadhense* MR193 (R) and MR226 (S)) on LJ slants, and after sufficient incubation, grown cells were collected and washed once with PBS. Apolar and polar lipids were extracted from the cell pellets using methods described by Dobson *et al.* (50). Polar lipids were analyzed by 2D-TLC using solvent system E, which is designed to separate phospholipids and LOSs (50). Glycolipids were visualized by charring following staining with either molybdophophoric acid (MPA) or alpha-napthol (for glycolipids).

### Diagnostic PCR markers for *M. riyadhense*

To develop diagnostic markers for *M. riyadhense* for distinguishing *M. riyadhense* from MTBC, *M. kansasii*, *M, szulgai* and *M. angelicum*, unique regions within the *M. riyadhense* reference genome compared to that of 152 other mycobacterial species were detected using Shustring (51). These regions were also examined by Proteinortho (34) and Blastn. *MR_00036*, *MR_00263*, *MR_00606* and *MR_01005* were selected as the amplification targets. Two primers for each gene were designed in this study:

MRDP-MR_00036-F (5′-TTCGTTGTCGGTTTCGTCGC-3′) and MRDP-MR_00036-R (5′-GCGTCAGCTCCACCGAAAAC-3′);

MRDP-MR_00263-F (5′-CCACCGCTGTTGGCGA-3′) and MRDP-MR_00263-R (5′-TTCGTCCCGTTGATCCCGTT-3′);

MRDP-MR_00606-F (5′-AACCTGCCCGATACGCACTT-3′) and MRDP-MR_00606-R (5′-ACTGTTCCTCCGTGGGGTTG-3′);

MRDP-MR_01005-F (5′-GACTGTGGGGTAACGGTGGA-3′) and MRDP-MR_01005-R (5′-CCGGTGATGTCGCCTACTCC-3′).

PCR was performed in a 25 μl reaction volume with 12.5 μl of GoTaq^®^ Green Master Mix (Promega, U.S.A.), 1 μl of 100 ng/μl gDNA, 1 μl with 10 nmol of forward and reverse primers, 3 μl of dimethyl sulfoxide (DMSO) and 19 μl of nuclease-free water. The PCR mixture was denatured for 5 min at 94°C; followed by 35 cycles of amplification involving a denaturation step at 94°C for 30 s, a primer annealing step at 59°C for 45 s and a primer extension step at 72°C for 45 s, and a final extension step at 72°C for 7 min. The ITS-F/mycom-2 primer set, which is a *Mycobacterium* genus-specific primer set, was used as a control, with amplification conditions as described previously (52). The products were electrophoresed in a 2% agarose gel for 60 min and visualized.

## RESULTS

### Assembly and annotation of the *M. riyadhense* genomes

Between April 2011 and March 2017, eight clinical cases of infection with *M. riyadhense* were recorded in male patients aged from 8 to 82 years. The comparison of different assemblies and genomic variations of all sequenced *M. riyadhense* strains is listed in Table 1. We obtained chromosomes of all eight isolates in single contiguous sequences for genome comparison at a high resolution. The circular nature of the chromosome and the circular plasmids (pMR01, pMR02 and pMR03) were demonstrated through Gepard (31).

**Table 1.** Comparison of *M. riyadhense* strains’ assemblies and genomic variations

	Type strain assembly GCA_002101845.1	MR193	MR206	MR210	MR222	MR226	MR244	MR246	MR1023
Assembly size (bp)	6 269 850	6 695 517	6 835 855	6 528 955	6 533 138	6 888 178	6 744 506	6 916 580	6 906 827
Chromosome size (bp)	6 269 850	6 129 600	6 288 531	6 034 715	5 960 707	6 243 587	6 258 360	6 289 824	6 306 178
Contigs	263	7	6	2	7	3	9	4	9
Gaps in chromosome	262	0	0	0	0	0	0	0	0
pMRLP	A	P	P	P	P	P	P	P	P
pRAW-like plasmid	A	P	A	A	P	P	A	A	A
CDS	5168	5901	6124	5800	5852	6077	6064	6060	6185

P: Presence; A: Absence.

In this study, we further identified the potential linear plasmids present in all 8 strains and circular plasmids (present in 3 strains as shown in Table 1) in *M. riyadhense* (Supplementary Figures S2 and S3). The pMRLP plasmids that are present in all 8 strains have no significant repetitive sequence that would support a circular topology and hence they likely represent linear plasmids. When compared with the circular plasmids of other species, such as pRAW in *M. marinum* (53), pMAH135 (54) and pMA100 (55) of *M. avium*, pMyong1 from *Mycobacterium yongonense* (56), pMK12478 (57) from *M. kansasii* and several plasmids from *Mycobacterium chimaera* (58), a high similarity was observed. The plasmid pMRLP01 in MR226 contains a pair of partitioning genes (*parA*/*parB*) that are involved in active segregation and thus stabilize the inheritance of the plasmid (59). The latter are known to contribute to genome evolution by active DNA transfer and exchange (60). The progressive alignments of the assembled chromosomes (Supplementary Figure S3A), pMRLP plasmids (Supplementary Figure S3B) and circular plasmids (Supplementary Figure S3C) of each *M. riyadhense* strain show that the chromosomes are relatively conserved; however, the likely linear plasmids present in all eight sequenced isolates are diverse from both structural and similarity perspectives, while the pRAW-like plasmids are present in only the MR226, MR193 and MR222 strains.

A previous study showed that *M. tuberculosis* genomes are highly conserved (61) in comparison to *M. canettii* (62). A much higher number of SNPs were detected in Smooth Tubercle Bacilli (STB) genomes compared to those of MTB strains (61,62). To study the genetic variations of the *M. riyadhense* strains, we called SNPs from all the strains and generated the SNP-based phylogeny using 43 136 polymorphic sites (Supplementary Figure S4). The SNP-based phylogeny revealed a group of five closely related *M. riyadhense* strains and three more distantly related strains, each of which is separated from the other strains by a long branch. The pair-wise comparison of SNPs between any 2 phylotypes is greater than the diversity between *M. tuberculosis* strains (61), while it is smaller than that seen within *M. canettii* strains (62), and the variation between the five closely related *M. riyadhense* strains is comparable to the SNP variation in *M. tuberculosis* strains. Currently, information on the genomic diversity of *M. riyadhense* strains is available only from a few isolates representing restricted geographic locations such as Saudi Arabia. Hence, we speculate that the current phylotype information may be subject to revision once genomic data become available for a larger cohort of *M. riyadhense* strains from wider geographic locations and ecological niches.

### Regions of difference (RDs) in *M. riyadhense*

The RDs were originally described as genomic regions present in virulent *M. bovis* and *M. tuberculosis* but absent from the *M. bovis* BCG genome (63). RD loci were subsequently described across the MTBC (64) and contain functions believed to contribute to pathogenicity (65–67) and may link to the evolution of MTBC species (68). *Mycobacterium riyadhense* was found to harbor most of the RD loci (RD1, RD3-R11, R13-RD16) that are also intact in *M. tuberculosis*, while 2 of the RDs show unique deletions, RD2^riyadh^ (Supplementary Figure S5A) and RD12^riyadh^ (Supplementary Figure S5B).

RD2 was originally described as deleted in *M. bovis* BCG vaccine strains. Subsequently, it was shown that disruption of RD2 in *M. tuberculosis* led to decreased proliferation *in vivo* and impaired modulation of the innate immune response (66). The RD2 locus in *M. riyadhense* also harbours a deletion, but one that is distinct from that in *M. bovis* BCG. It is a larger deletion than the originally described RD2^BCG^ as RD2^riyadh^ contains 29 genes (*M. tuberculosis* coordinates were used, *rv1971*∼*rv2000*, location 2,216,498∼2,246,766); eight genes within this locus (*M. tuberculosis* coordinates were used*, rv1978*, *rv1979c*, *rv1980c*, *rv1981c*, *rv1983*, *rv1984*, *rv1987*, *rv1988*) have orthologs elsewhere in the *M. riyadhense* genome (*MR_05764*, *MR_05852*, *MR_02310*, *MR_02993*, *MR_00486*, *MR_02995*, *MR_02325*, *MR_02349*, *MR_01747*), suggesting possible functional redundancy.

The RD12 locus shows deletions across MTBC members, including *M. tuberculosis* (69), *M. bovis* (70), *Mycobacterium caprae* and *M. orygis* (71). *Mycobacterium canettii* isolates (except group B (72)) also show an independent deletion at the RD12 locus named RD12^can^ (3,479,430∼3,491,866, *rv3111*∼*rv3126*), which is distinct from RD12^bovis^ (3,484,740∼3,487,515, *rv3117*∼*rv3121*). We identified another unique deletion at the RD12 locus in *M. riyadhense*, designated RD12^riyadh^, which encompasses a larger region than RD^can^ and RD12^bovis^, encompassing *rv3108*-*rv3127* (3,477,171∼3,492,150) (Supplementary Figure S5B). It is intriguing that multiple mycobacteria show independent deletion events at the RD2 and RD12 loci, suggesting selective forces may play a role in this variation.

### Comparative phylogeny of *M. riyadhense* with other Mycobacteria

**Figure 1.**
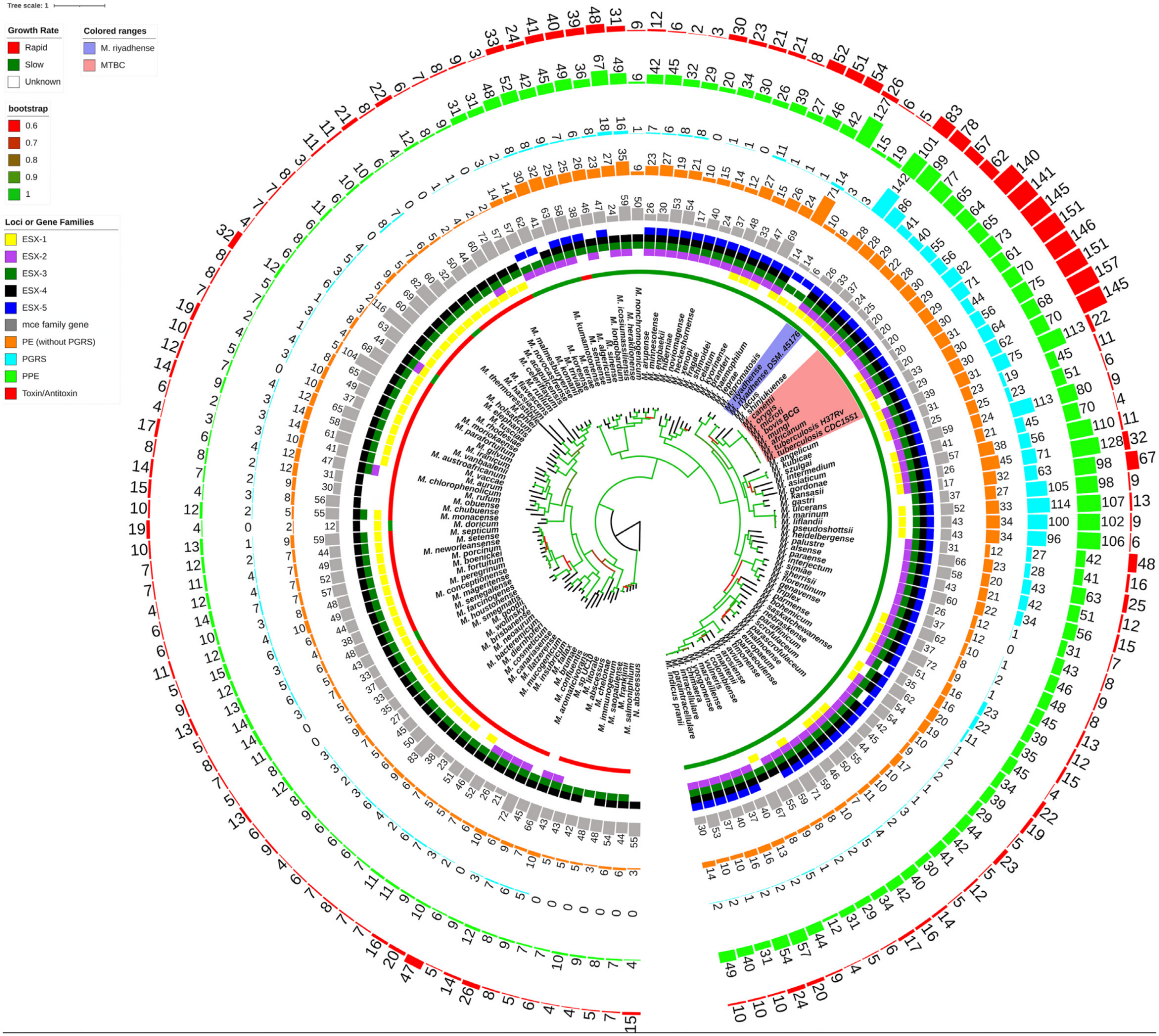
A phylogenetic tree of 147 mycobacterial genomes showing close relationship of *M. riyadhense* and MTBC. The phylogeny was constructed using 147 available genomes by concatenating and aligning amino acid positions across 400 shared proteins automatically identified in the chosen genomes by PhyloPhlAn2 (35). Coloured shades highlight the *M. riyadhense* strains and MTBC. We annotated the growth rate, presence/absence of ESX systems (based on homology analysis), the number of annotated gene family members, the *M. tuberculosis* T/A orthologs for each strain external to the phylogeny. Branches are coloured based on the bootstrap values as shown in the legend in the left. The colour code of the gene families, ESX systems, growth rate and bootstrap range is listed on the left side figure legend.

The phylogenetic tree shows that the slow-growers and rapid-growers are separated into two different clades and that the early branching species are fast-growers (Figure 1). The overall topology of our tree is similar to that of previously published phylogenetic trees (73–75); however, there are a number of features in our analysis that are worth highlighting. Hence, our current phylogeny comprises a large set of mycobacterial taxa (147 genomes) and was inferred with a robust phylogenetic approach (maximum likelihood-based phylogeny) using a substantially larger set of amino acid sequences to previous studies. By way of comparison, Tortoli *et al.* generated their phylogeny with a matrix of ANI-divergence scores (73), which is not based on specific evolutionary models and is less reliable than the maximum likelihood-based phylogeny used here. Furthermore, while Sapriel and Brosch used a similar methodology to the one we employed, they used a smaller data set (75). The phylogenetic analysis by Fedrizzi *et al.* (74) is similar in scale and methodology to that in the current manuscript, although our approach was broader. Given these improvements, our current study provides a strong confirmation of prior results and reveals several new perspectives.

Our phylogenetic analysis shows that *M. riyadhense* along with *M. lacus*, *M. shinjukuense* and MTBC form a monophyletic group that has descended from a common ancestor. Tortoli *et al.* found that *M. riyadhense*, *M. lacus*, *M. shinjukuense*, *M. decipiens*, *M. szulgai*, *M. angelicum* and MTBC form a monophyletic group (74) which differs from our findings that *M. szulgai, M. angelicum* are from the sister group. A wide evolutionary gap between the *M. leprae* clade and MTBAP in our phylogenetic analysis contradicts Fedrizzi *et al.* (74), where they showed the most recent common ancestor of the MTB and *M. leprae* clade was more recent than the MRCA of MTB and the MTBAP clade. *Mycobacterium riyadhense* is located within the same clade as obligate and opportunistic mycobacterial pathogens that include the MTBC, *M. marinum*, *M. kansasii*, *M. leprae* and related host-restricted mycobacteria with reduced genomes and decreased survivability in the environment.

The PE/PPE and *mce* family genes are known to be important for host adaptation (76) and pathogenicity (77). We observed an expansion of PE/PPE family genes in the MTBC members but also in *M. riyadhense* MR226 and other pathogenic species, such as *M. kansasii* and *M. ulcerans*. The number of *mce* family gene orthologs has not significantly changed across mycobacterial genomes (Figure 1). Our results agree with previous findings that during their evolution, the ESX systems were derived from the ancestor ESX-4, as shown in Figure 1 at the root node, and then ESX-3, ESX-1, ESX-2 and ESX-5 evolved by horizontal transfer (78).

**Figure 2.**
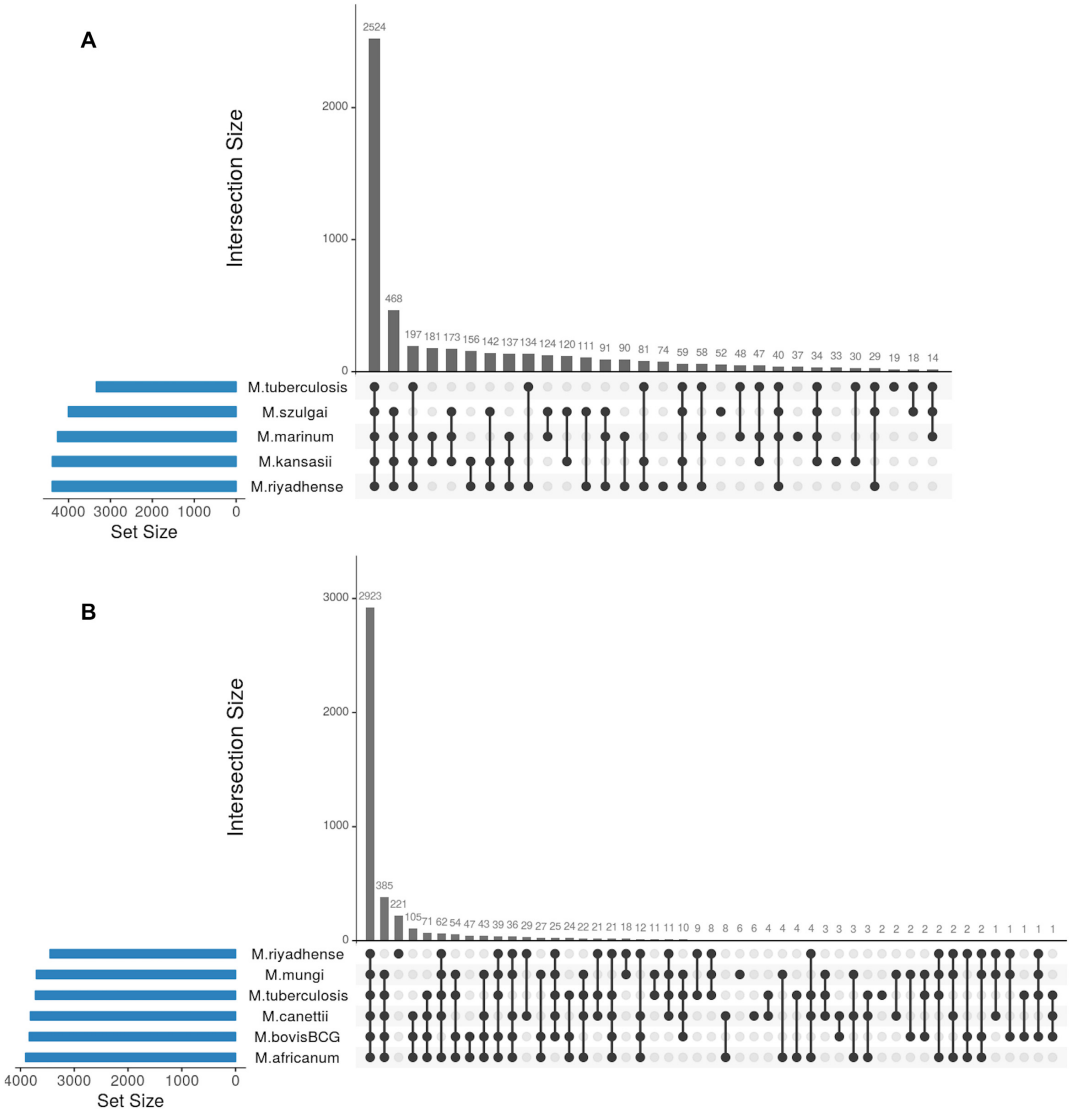
UpSet plot showing the comparison of orthologous groups. UpSet plot showing the orthologous groups total set size and overlaps between the (**A**) *M. riyadhense*, *M. tuberculosis* H37Rv, *M. marinum* M, *M. kansasii* 12478 and *M. szulgai* and (**B**) *M. riyadhense* MR226 and five species within the MTBC.

*M. riyadhense* shares a larger number of orthologs (3,122) with *M. tuberculosis* than with *M. kansasii* (2,978 orthologs), *M. marinum* (2,962 orthologs) and *M. szulgai* (2,724 orthologs) amongst the NTM that are closely related to the MTBC (Figure 2A). A total of 134 orthologs are exclusively shared between *M. riyadhense* and *M. tuberculosis*, while the number of orthologs exclusively shared between *M. tuberculosis* and *M. kansasii* (30 orthologs), *M. marinum* (48 orthologs) and *M. szulgai* (18 orthologs) is less (Figure 2A). It is notable that 34 T/A genes are shared exclusively between *M. riyadhense* and *M. tuberculosis* within this comparison.

The comparative analysis of the orthologous groups of *M. riyadhense* and the MTBC is informative. Firstly, 385 orthologous groups present across all MTBC species are absent from *M. riyadhense*. Secondly, 221 protein groups uniquely present in *M. riyadhense* are not found amongst the MTBC (Figure 2B). This latter group of *M. riyadhense* unique protein groups are likely required for *M. riyadhense* to maintain a broad functional repertoire so as to secure its survival. In contrast, the MTBC species have lost genes involved in free-living survival due to their obligate pathogen lifestyle.

**Figure 3.**
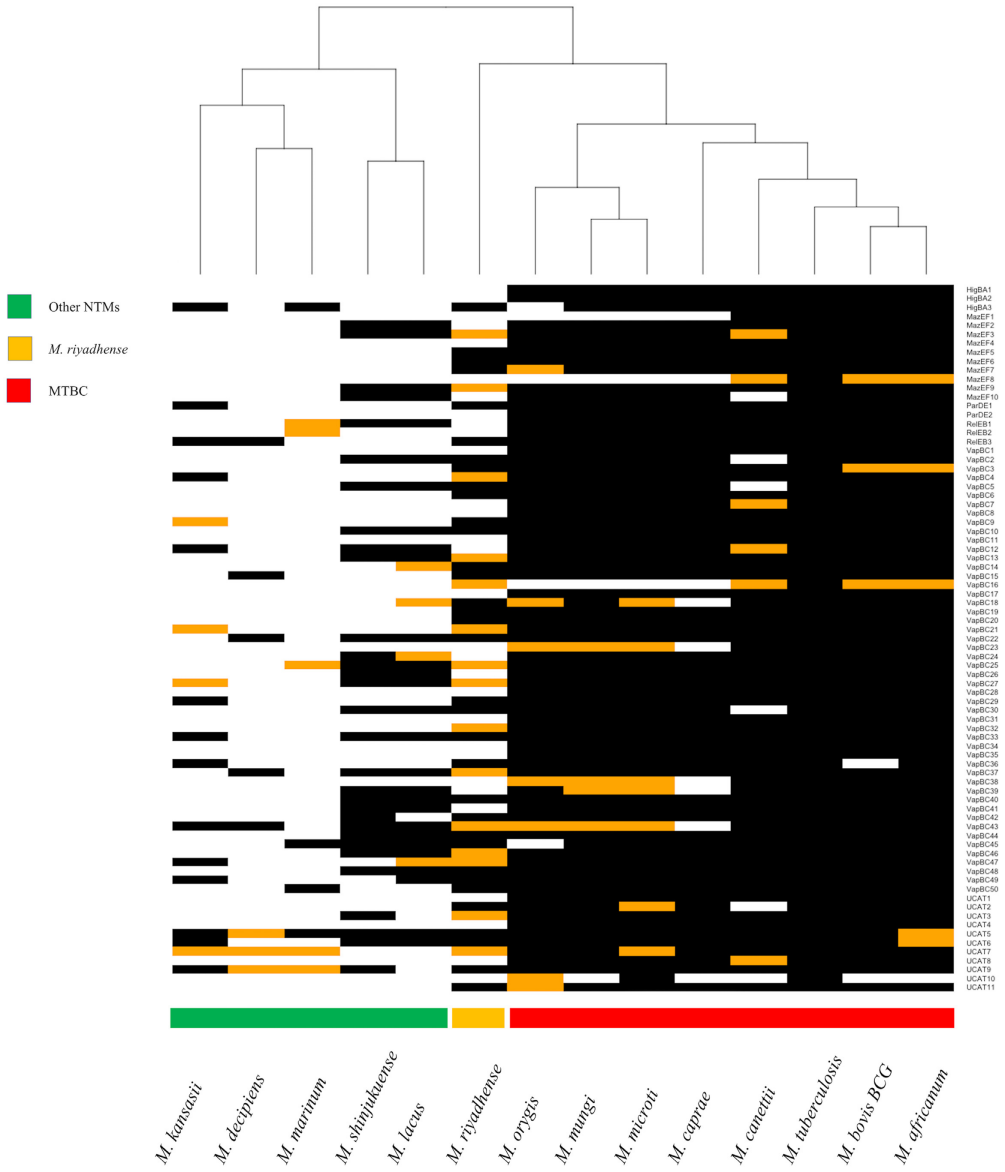
A hierarchical clustering of the presence (black) and absence (white) of *M. tuberculosis* H37Rv toxin/antitoxin orthologs in *M. riyadhense*, *M. marinum*, *M. kansasii*, *M. shinjukuense*, *M. lacus*, *M. decipiens* and MTBC species. The orange blocks denote the presence of either the toxin or antitoxin ortholog in a given pair of the T/A system. The black and white blocks represent presence and absence respectively. The name of the T/A system are shown for each row on the right.

We compared the 79 pairs of T/A systems (belonging to the HigAB, MazEF, ParDE, RelEF, VapBC and UCAT families) in *M. tuberculosis* with the T/A pairs found in other members of the MTBC and NTMs. We observed an expansion (83 out of the 158 orthologs) of the T/A orthologs (Figures 1 and 3) in *M. riyadhense* MR226 strain compared to other NTMs including *M. lacus, M. shinjukuense* and *M. decipiens*. A detailed comparison of the T/A *M. tuberculosis* H37Rv orthologs across *M. riyadhense* strains is illustrated in Supplementary Figure S6.

### *M. riyadhense* strains produce a distinct pattern of LOSs

**Figure 4.**
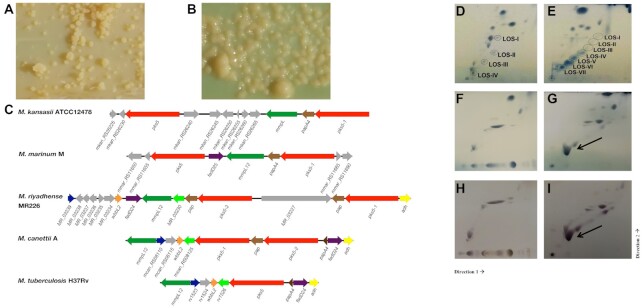
*pks5* loci in *M. riyadhense* and other related mycobacteria and 2D-TLC analysis of polar lipids extracted from selected *M. riyadhense* strains, *M. kansasii* (subtype I), *M. marinum* M, *M. smegmatis* MC-1551. (**A**) Rough-dry colony morphology (MR193) and (**B**) smooth morphology (MR226) of *M. riyadhense*. (**C**) Genetic locus map of the *pks5* gene cluster from *M. riyadhense* MR226, *M. marinum* M (partial), and *M. kansasii* ATCC12478 (partial), *M. canettii* A and *M. tuberculosis* H37Rv (drawn to scale). The arrows show the direction of transcription and the genes are coloured according to the orthologous relationships. *Mycobacterium tuberculosis* H37Rv does not produce LOSs and the *pks5* loci was drawn for genomic comparison purpose. Polar lipids from two known LOS producers, *M. marinum* (**D**) and *M. kansasii* (**E**), are included to illustrate the migration pattern of LOS species in System E. (**F**) 2D-TLC analysis of polar lipids extracted from select *M. riyadhense* rough strain or smooth (**G**) strain. A separate staining with alpha-napthol also confirmed that this was a glycolipid species from the same (**H**) rough and (**I**) smooth strain. (D**–**G) were charred after staining with MPA, while (**H**) and (**I**) were charred after staining with alpha napthol. LOS III from *M. riyadhense* is indicated by a solid arrow.

It is well-known that the smooth morphotypes of mycobacteria, such as *M. avium* (79), *M, abscessus* (80) and *M. kansasii* (13), are less virulent than the rough morphotypes. It is noteworthy that we observed both smooth (MR210, MR222, MR226, MR244, MR246 and MR1023) (Figure 4A) and rough (MR193 and MR206) (Figure 4B) morphologies in *M. riyadhense* strains. The lipooligosaccharides are an important class of glycolipids that have been linked to diverse mycobacterial phenotypes including colony morphology. The LOS-biosynthesis gene locus has been characterised in *M. marinum* (14), *M*. *kansasii* and *M*. *canettii* (81). We therefore sought to first examine whether the genetic machinery for the production of LOS is present in the *M. riyadhense* genome, and then to follow up on the genome-level predictions with lipid analyses of rough and smooth variants using thin-layer chromatography (TLC).

To investigate the genetic basis for the smooth and rough morphology in the *M. riyadhense* strains, we compared the LOS locus of the eight strains (Supplementary Figure S7A and Supplementary Table S3). We compared the locus map of the eight strains and have found several variations in the rough strains: (1) the LOS locus maps are the identical for strains MR226, MR246 and MR1023, which are all smooth strains; (ii) the *fadD24* gene is intact except in strain MR206 (rough) (Supplementary Figure S7B) where a transposable element fragment was detected; *fadD24* is a fatty-acid-AMP synthetase; a mutation in the *fadD24* ortholog in *M. marinum* (*fadD25*, *mmar_2341*) generated a rough morphology, which matches our genotype prediction (14); (iii) absence of *MR_03241* in both smooth (MR210) and rough (MR193) strains (Supplementary Figure S7A,S7C); (iv) a 23-gene insertion (several transposases, integrase and IS elements were detected in this region) was found in the rough strain MR193 (Supplementary Figure S7D), between *MR193_03124* and *MR193_03148*. *MR_03251*, which is a glycotransferase without any orthologs in *M. canettii*, *M. tuberculosis* or *M. marinum* is missing in the LOS locus of MR193. Analysis of LOS profiles via mutagenesis/complementation of *MR_03251* in *M. riyadhense* will help to confirm the function of this gene.

The *pks5* locus is involved in polyketide metabolism and previous study has shown this locus regulates the S/R morphology of *M. canettii*. The *pks5* and *pap* genes in the LOS locus are intact in *M. riyadhense*, as is the case in *M. canettii*, but not in *M. tuberculosis*, where the former is truncated and the latter deleted (81). Remarkably, the *M. riyadhense pks5* gene locus layout is dissimilar to that in *M. canettii*, *M. tuberculosis*, *M. kansasii* and *M. marinum* (Figure 4C). A previous study speculated that *wecE* is involved in the biosynthesis of the unusual sugar residue of LOS-IV and a *wecE* mutant cannot generate LOS IV in *M. marinum* (14). We observed that the *wecE* and *galE6 M. marinum* orthologs are absent from the *M. riyadhense* genome (Supplementary Table S2). The same study also showed that a *galE6* (UDP-glucose 4-epimerase) mutant was also not able to produce LOS II. Thus, absence of *wecE* and *galE6* is likely to cause an accumulation of LOS II* and the lack of fully formed LOS IV, which have previously been shown to increase the pathogenicity of *M. marinum* (14).

To correlate rough versus smooth colony morphology with LOS production, we extracted polar lipids from the strains and analysed them by 2D-TLC using solvent system E (50), which is designed to separate phospholipids and LOSs. Charring of the TLC plates with alpha-napthol revealed glycolipids, including the accumulation of a species that migrated at a position similar to that of LOS III. This lipid was seen only in smooth strains; species with migration patterns similar to LOS I and LOS II were observed, while no spots were observed where LOS IV is expected to migrate. This result was not unexpected because all *M. riyadhense* strains lack *wecE* ortholog, which is required for the extension of LOS II to LOS IV (Figure 4). Additionally, the relative levels of the predominant LOS species in *M. riyadhense* seem to be quite high when compared to those seen in other LOS-producing mycobacteria (Figure 4D, E). Conversely, the rough strains did not produce any glycolipids that migrated in the positions corresponding to LOSs (Figure 4F, H).

### *PE-PGRS33* locus

The *pe-pgrs33* (*rv1818c*) locus encodes the exported protein PE_PGRS33 that plays an important role in the pathogenesis of *M. tuberculosis* (82). A previous study (83) showed that *pe-pgrs33* is present in all MTBC members but not in *M. canettii*, which implies a specific *pe-pgrs33* insertion event in the ancestor of MTBC strains. In this study, we performed a comparison of the genomic context of the *pe-pgrs33* locus using the Artemis Comparison Tool (ACT) (Supplementary Figure S8A), and precisely identified the breakpoint in the *M. riyadhense* MR226 genome (3,155,129bp-3,156,672bp, within the *MR_02789* reading frame) and the MTB H37Rv genome (2,061,129bp-2,062,831bp). *MR_02789* also contains orthologs in *M. marinum* M (*mmar_2693*) and *M. kansasii* ATCC12478 (*mkan_RS00295*). The overall synteny in the locus in MR226 also shares a high similarity with *M. canettii PE-PGRS33 locus*, and it is evident that the lactoylglutathione lyase gene (*MR_02789*) ortholog was lost before the MTBC speciation (Supplementary Figure S8B).

### Type VII secretion system of *M. riyadhense*

All five ESX systems (ESX1-ESX5), which are Type IV Secretion (T7S) systems, were found in the *M. riyadhense* strains (Supplementary Figure S9) with the same level of synteny except for *espK* which was truncated in MR206 (Supplementary Table S4). The overall gene arrangement of the ESX1-ESX5 loci is similar in both *M. riyadhense* and *M. tuberculosis* (Supplementary Figure S9) (84). The *espACD* operon, which is essential for secretion of virulence factors via ESX1, and *eccB* and *eccC* genes in ESX-2, are absent from the ESX-2 system in *M. riyadhense* compared to *M. tuberculosis* H37Rv. This conserved synteny reinforces the previous results of phylogenetically relatedness of *M. riyadhense* to MTBC. As noted before, the pMR01-03 plasmids also contain an extra ESX-P5 locus, which could indicate a role for this plasmid in mediating pathogenicity (Supplementary Figure S2).

### Comparative transcriptional response of murine macrophages upon infection by *M. riyadhense*, *M. tuberculosis*, *M. kansasii* and *M. bovis* BCG

Our genomic analysis of *M. riyadhense* revealed a range of genes and potential gene networks that could play a role in host–pathogen interactions. We therefore sought to assess the initial interaction of *M. riyadhense* with macrophages, using the murine RAW264.7 cell line as our experimental model. As comparator strains in our analysis, we performed parallel infections with *M. kansasii*, an opportunistic pathogen that also contains an orthologous ESX-1 system (Figure 1), *M. bovis* BCG and *M. tuberculosis*. These comparisons allowed us to explore the *M. riyadhense*-triggered innate immune responses.

To compare the innate immune responses of macrophages infected with these mycobacterial isolates, their transcriptional profiles were analysed at 3, 24 and 48 h post infection (hpi) compared to uninfected controls. In total, 1496 genes, or 3.79% of the genes in the genome (39 504 genes in total), were upregulated when comparing infected cells to non-infected cells at each time point (Tables S5-S16; Log_2_Fold Change> = 2, padj<0.01).

**Figure 5.**
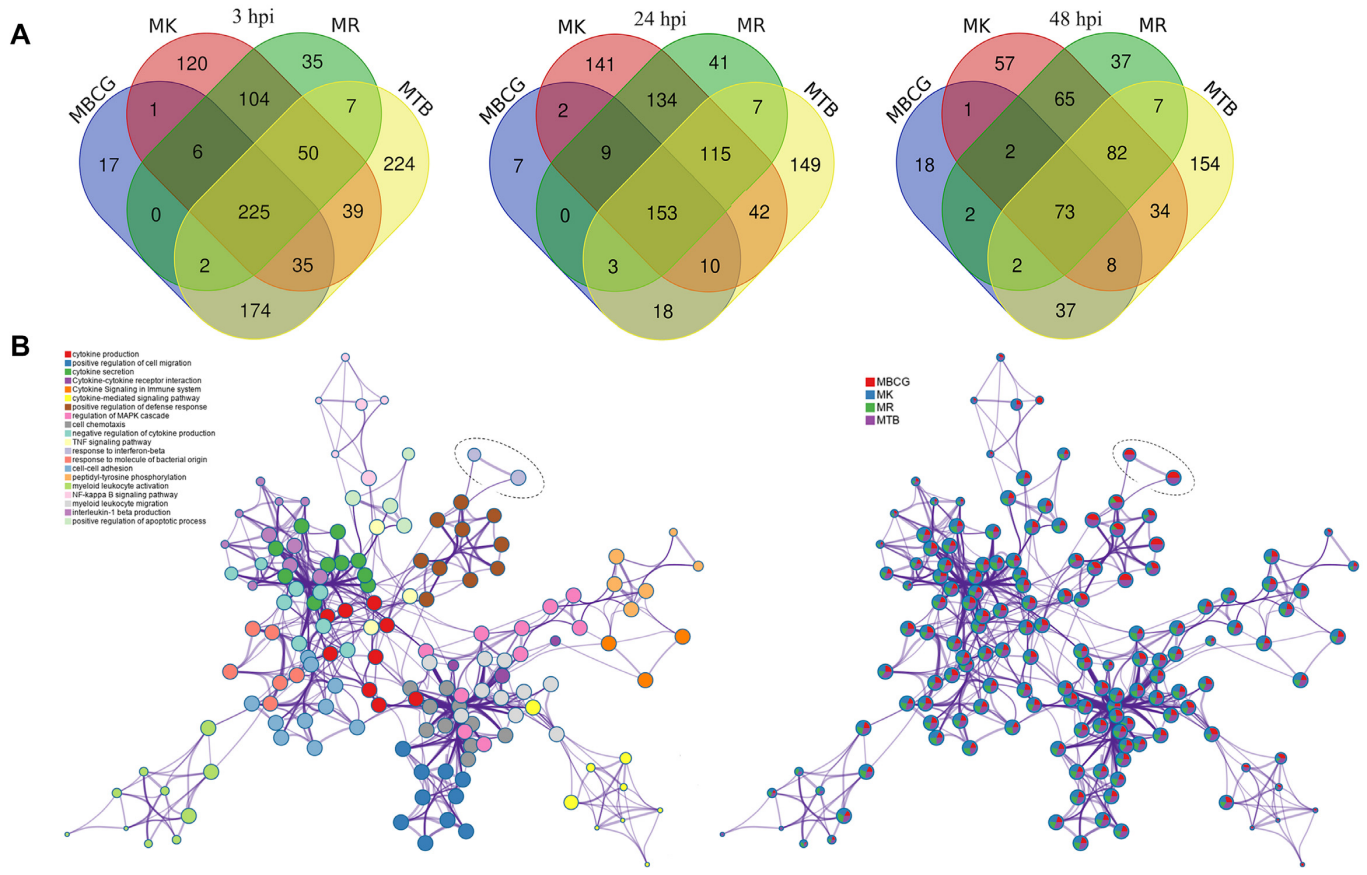
The transcriptional response of RAW264.7 macrophages to mycobacterial infections. (**A**) Venn diagram showing the overlap of differentially expressed genes upon *M. riyadhense* (MR), *M. kansasii* (MK), *M. bovis* BCG (MBCG) and *M. tuberculosis* (MTB) infection over 3, 24 and 48 hpi, and (**B**) Network of enriched terms for each bacterial infection upon 3 hpi. Each node represents an enriched term and the size of the circle represents the number of genes within that term as applied by the Metascape (49). Terms with a similarity > 0.3 are connected by edges. The left panel is coloured by cluster, while the right panel is colour-coded based on different bacterial infections. The nodes that represent the ‘response to interferon-beta’ are highlighted by a dotted circle.

A large number of DE genes overlapped at each timepoint: 225, 153 and 73 (Figure 5A). These genes are responsible for general immune responses, such as proinflammatory genes associated with TLR signalling (e.g., upregulation of *Il-1β*, *Tnf-α*, *Ccl4*, *Ptgs2* and *Cxcl2*, albeit to different absolute levels; Tables S5, S8, S11, S14) and cytokine activity-related genes (upregulation of *Ccl7*, *Ccl2*, *Cd86*, *Ccl22*, *Il-1β* and *Ccl3*). Genes uniquely triggered by *M. riyadhense* infection compared with other mycobacteria included *Cd22*, *Gpr65*, *Adh7* and *TrnT* (Supplementary Table S11); however, no functional categories were statistically enriched from this category of genes.

The largest number of DE genes were identified from *M. tuberculosis* infected RAW264.7 cells 3 hpi, with 756 genes being upregulated. As expected, the transcriptional responses due to *M. riyadhense* and *M. kansasii* infections clustered more closely while the BCG and *M. tuberculosis* responses clustered together (Supplementary Figure S10). One of the key differences in the macrophage transcriptional responses between *M. riyadhense* or *M. kansasii* and *M. bovis* BCG or *M. tuberculosis* pairs at 3hpi was the differential induction of Type I IFN signaling-related genes (Figure 5B and Supplementary Figure S11A). The expression of *Ifnb1* was relatively lower in macrophages infected with *M. riyadhense* or *M. kansasii*, and that of IFN-β-controlled genes was only significantly enriched with, or more strongly upregulated by, *M. bovis* BCG or *M. tuberculosis* (Figure 5B).

In addition, activation of the TLR4/Myd88/ NF-κB signalling axis triggers expression of *Tnf-α*, *Il-1β* and *Cox-2*. These genes were potently induced by all four species, but less by *M. riyadhense* (Supplementary Figure S11B), suggesting that *M. riyadhense* triggers reduced signalling through TLR4. We also observed that *M. riyadhense or M. kansasii* were stronger activators of Liver X Receptors (LXR), compared to *M. bovis* BCG or *M. tuberculosis* (Supplementary Figure S11B). This is shown by the relatively higher expression of *Abca1* and *Abcg1* in *M. kansasii* or *M. riyadhense* which could be a secondary effect of a less potent induction of NF-κB pathway (85).

### Developing a rapid PCR-based diagnostic marker for *M. riyadhense*

Due to the issues previously encountered in diagnosing *M. riyadhense* infections (4,7), correct and prompt identification of cases upon presentation at healthcare units is of paramount importance. We therefore sought to translate our knowledge on the genome sequences into a PCR diagnostic test that could be used in a clinical microbiology setting to distinguish *M. riyadhense* from other mycobacteria, including the members of the MTBC.

By identifying unique K-mers ranging in size from 11 bp to 4209 bp (Supplementary Figure S12) in the assembled genome compared to the genomes of 152 other mycobacterial species, four primer sets were developed targeting the *MR_00036*, *MR_00263*, *MR_00606* and *MR_01005* genes. The MRDP primer pair MRDP-F/MRDP-R amplified a single product from each of the eight isolates of *M. riyadhense* (Supplementary Figure S13) but not from other mycobacterial species, including *M. tuberculosis*, *M. bovis*, *M. kansasii*, *M. marinum*, *M. szulgai*, *M. avium* and *Mycobacterium angelicum*. This result shows that the MRDP-F/MRDP-R primers are highly specific to *M. riyadhense* and form the basis for a simple diagnostic PCR that can inform appropriate treatment protocols.

## DISCUSSION

*Mycobacterium riyadhense* has become a clinically relevant NTM species globally (4–7,9,10). Contrary to prior publications on *M. riyadhense* that have been based primarily on clinical case reports, here we present the largest and most comprehensive genomic study undertaken to date on clinical *M. riyadhense* isolates. The eight new *M. riyadhense* strains sequenced in this study originated from pulmonary infections, with some having additional extra-pulmonary involvement, fulfilling the American Thoracic Society/Infectious Diseases Society of America (ATS/IDSA) criteria for NTM infection (86).

It is well known that plasmids are important ‘vehicles’ for the exchange of genetic material between bacteria or between chromosomes and extra-chromosomal plasmids. In this study, we identified likely linear plasmids pMRLP (present in all 8 strains) and circular plasmids (present in 3 strains as shown in Table 1) in *M. riyadhense* (Supplementary Figures S3C and S4). Linear plasmids were first described in 1989 in maize (87) and have also been found in *Actinomycetales* and *Mycobacterium* species, such as *Mycobacterium xenopi*, *Mycobacterium branderi* and *Mycobacterium celatum*. They are often accompanied by a circular plasmid in the same host (88).

The pRAW-like circular plasmids (pMR01-03) identified in this study all harbour both a T4S (Type IV secretion system) and T7S systems, which are necessary for conjugation (53), and facilitate the exchange of genetic material between different species of slow-growing mycobacteria. We therefore speculate that pMR01-03 are likely novel conjugative plasmids.

Our comparative analysis of *M. riyadhense* genomes with the MTBC and a large collection of NTMs provide unequivocal evidence that *M. riyadhense* is one of the close known NTM species to the MTBC and forms a phylotype with *M. lacus* and *M. shinjukuense*. Indeed, while our manuscript was in preparation, independent work by Sapriel and Brosch (12) also showed the close phylogenetic relationship of *M. riyadhense* to the MTBC, suggesting that it forms part of an MTB-associated phylotype. Our analyses of multiple *M. riyadhense* isolates complements and extends the findings of Sapriel and Brosch by revealing that expansion of T/A pairs, modification of secretion systems, alterations in cell wall lipids all play key roles in the evolution of the MTBC.

Our study shows that *M. riyadhense* shares a larger number of orthologs with *M. tuberculosis* than *M. kansasii* and *M. marinum*, notably in the T/A gene family (Figures 2 and 3). Eighty-three T/A orthologs were found in *M. riyadhense* MR226, far greater than the number of orthologs observed in any of the other NTMs (Figures 1 and 3). The expansion of T/A genes among the MTBC offers additional evidence that suggests that the original acquisition of T/A modules into mycobacteria through lateral gene transfer played a key role in the development of pathogenicity (89,90). The other virulence-associated gene family compared in this study is the *mce* family. The number of *mce* gene orthologs show the plasticity of this gene family. A recent study has shown that the *mce* homology between rapid and slow-growing species was low (<50%), which may indicate a possible adaptation of *mce* homologs to an intracellular lifestyle in slow-growing mycobacteria (91).

*Mycobacterium riyadhense* strains appeared as both smooth and rough colony forms when grown on solid LJ media. The observation of smooth and rough colony variants is seen in other mycobacterial pathogens where it is linked to presence or absence of LOS. For example, the presence or absence of LOS from *M. canettii* causes a transition from smooth to rough colony variants, respectively, with rough variants showing increased virulence. MTBC strains lack LOS, and it has been suggested that the removal of LOS was a key event in the evolution of the MTBC species towards their current obligate pathogen status (81). In *M. riyadhense* smooth colony variants, we observed the presence of LOS I and LOS II but the absence of LOS IV. These biochemical observations agree with the genomic prediction that *M. riyadhense* strains lack a *wecE* ortholog, which is required for the extension of LOS II to LOS IV in *M. marinum* (14). Overall, our results show that *M. riyadhense* exhibits a LOS production phenotype distinct from that of other LOS-producing mycobacteria. The truncated *fadD24* gene in MR206 and absence of glycotransferase *MR_03251* is likely responsible for the rough morphology of MR193 and MR206 strains, but further complementation experiments are required to provide a definitive confirmation.

The T7S systems are key elements of mycobacterial virulence. All the 5 ESX T7S systems present in *M. tuberculosis*, and known to be involved in virulence and pathogenicity, were found in *M. riyadhense* with very similar gene arrangement. An additional ESX-P5 system was also found on the circular plasmid pMR01-03. pRAW-like plasmids have been shown in many different NTMs, including *M. kansasii*, *M. marinum*, *M. chimaera* and *M. avium*. The extensive presence of these plasmids may also explain the distribution of the T7S system during the evolution of the mycobacterial genus (53).

Differences in macrophage gene expression after infection with the mycobacterial strains used were most evident at 3hpi which is likely reflective of the initial macrophage response to infection. In support of this, the comparative transcriptomic response of macrophages after infection with *M. riyadhense* or *M. kansasii* versus *M. bovis BCG* or *M. tuberculosis* pairs showed very similar signatures, suggesting that the bacterial cell wall determinants, key determinants of the initial interactions these bacterial pairs with host cell receptors are well conserved.

We observed an increased abundance of *Ifnb1* mRNA levels in all of the four Mycobacterial species (log_2_FC, *M. kansasii*: 7.1; *M. riyadhense*:5.3; *M. tuberclosis*:8.2; *M. bovis* BCG:7.4) at 3 hours post infection. However, *Ifnb1* expression levels were comparatively lower in the *M. riyadhense/M. kansasii* pair in comparison to the *M. bovis BCG/M. tuberculosis* pair. It has been shown that *M. kansasii* and *M. fortuitum* induce stronger IFN-β responses in comparison with *M. tuberculosis* after infection of bone marrow-derived dendritic cells (BMDCs) at MOI of 10: 1 (92), while this was not observed in infection of the RAW264.7 cells in our study. This could be due to differences in the experimental systems used, or a post-transcriptional regulatory mechanism of *Ifnb1* transcripts that modulates protein levels. Type I IFNs can have either beneficial or detrimental consequences in bacterial infections (93). While generally promoting the growth of *M. tuberculosis* they may have an opposite effect on NTMs (94), whether they have a protective or detrimental role in *M. riyadhense* infection remains to be addressed.

The clinical presentation of our cases was by and large indistinguishable from disease caused by *M. tuberculosis*, as reported earlier (7), but with a negative *M. tuberculosis* PCR. Due to the relatively recent emergence of *M. riyadhense* as an important clinical pathogen coupled with its misdiagnosis as *M. tuberculosis* by commercially available kits, we developed an accurate set of diagnostic markers based on the genomic datasets generated in this study. The primer sets accurately detect *M. riyadhense* in a mixed cocktail of closely related mycobacteria and can hence serve as part of an accurate and fast diagnostic protocol in clinical settings thus reducing the need for strict isolation, laborious contact tracing and inappropriate use of TB antimicrobials. The diagnostic primers are of immediate utility in distinguishing between MTBC and *M. riyadhense* and provide a basis towards developing a set of primers that can be used in broader epidemiological surveys. Our work, and that of previous studies, was based on *M. riyadhense* human clinical isolates. As only two studies have found the presence of *M. riyadhense* in water and soil without confirmation by bacterial culture, its environmental reservoir remains largely unknown. We believe that systematic screening of relevant environmental samples with the MRDP established in this study may help to identify the natural habitat of this bacterium and hence the source of human infections.

In conclusion, our study provides new insights into the evolution of the MTBC relative to extant NTMs, unveiling the genomic shifts from NTMs to MTBC before the speciation of the common ancestor of MTBC. Our work provides the underpinning data to support the use of *M. riyadhense* as a novel mycobacterium for the study of evolution, virulence and pathogenesis in the MTBC.

## DATA AVAILIBILITY

The *M. riyadhense* dataset is available at European Nucleotide Archive (ENA) under the study accession no. PRJEB32162. The derived datasets, including phylogenetic trees, *M. tuberculosis* T/A orthologs, protein orthologs comparison between 8 *M. riyadhense* strains etc. have been deposited in Zenodo (10.5281/zenodo.4434791).

## Supplementary Material

lqab070_Supplemental_Files

## Notes

Present address: Musa Garbati, Infectious Diseases Unit, Department of Medicine, University of Maiduguri, Nigeria.

Present address: Anwar Hoosen, Pathcare/Vermaak & Partners Pathologists, Pretoria, South Africa.
