# Supplementary material for: Insights into the ancestry evolution of the Mycobacterium tuberculosis complex from analysis of Mycobacterium riyadhense
Source: NAR Genom Bioinform. 2021 Aug 11;3(3):lqab070. doi: 10.1093/nargab/lqab070 (PMC8356964; doi:10.1093/nargab/lqab070)
Supplement: lqab070_Supplemental_Files [file lqab070_supplemental_files.zip › NAR_GAB_Suppl_R2.pdf]

Supplementary Figures

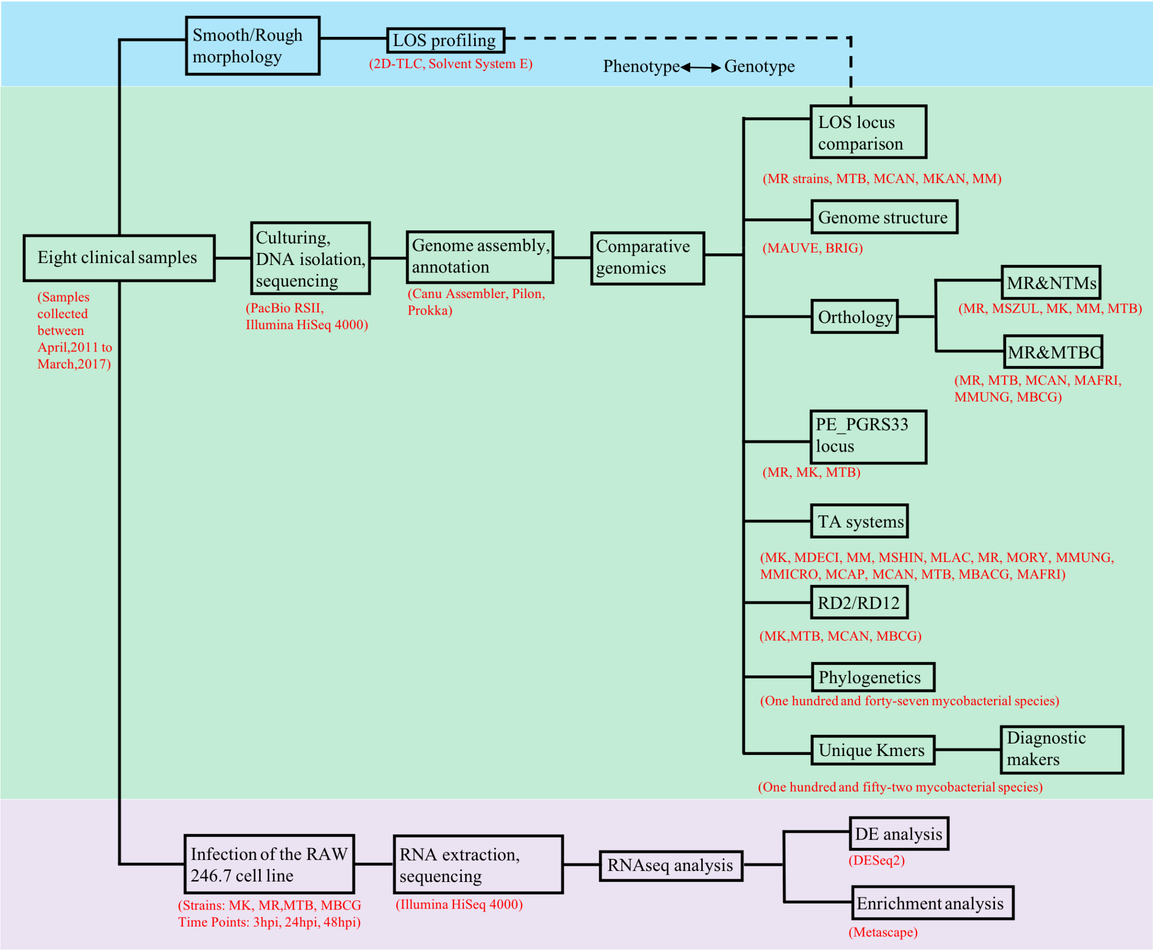

Figure S1: Flow chart showing the study design.

The programs, platforms, methods, or datasets used for each step are labeled at the bottom of each step. The background colors represent different aspects of the study design: blue represents the metabolites (lipooligosaccharides) profiling part, green represents the comparative genomicspart, and purple represents the transcriptome (host immune responses) part. The species abbreviations for this figure: MR: *M. riyadhense*; MTB: *M. tuberculosis*; MCAN: *M. canettii*; MM: *M. marinum*; MSZUL: *M. szulgai*; MK: *M. kansasii*; MAFRI: *M. africanum*; MMUNG: *M. mungi*; MBCG: *M. bovis* BCG; MDECI: *M. decipiens*; MSHIN: *M. shinjukuense*; MLAC: *M. lacus*; MORY: *M. orygis*; MMCIRO: *M. microti*; MCAP: *M. caprae*; The detailed strain information used in this study are available in Table S1.

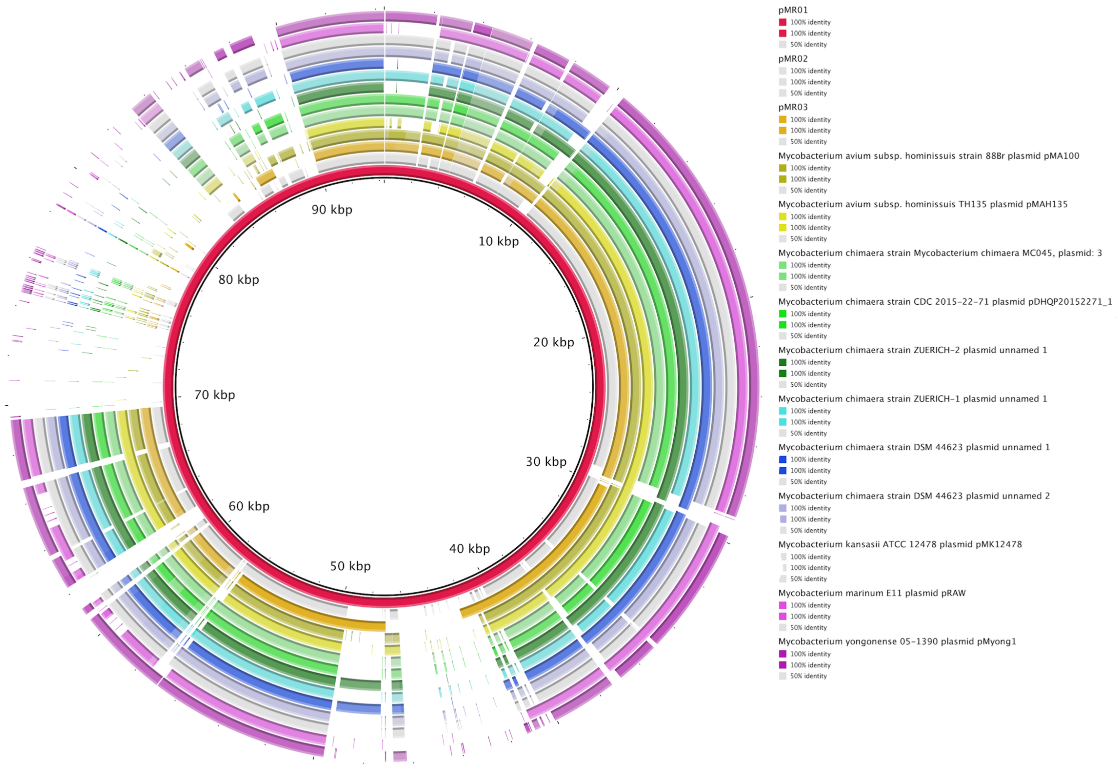

Figure S2: Circular map of the pRAW-like plasmids in *M. riyadhense* (pMR01, pMR02 and pMR03).

The circles show from outside to inside (1-14) BlastN similarity results against the pMR01 of various *Mycobacterial* plasmids. The corresponding plasmids used are listed on the right panel.

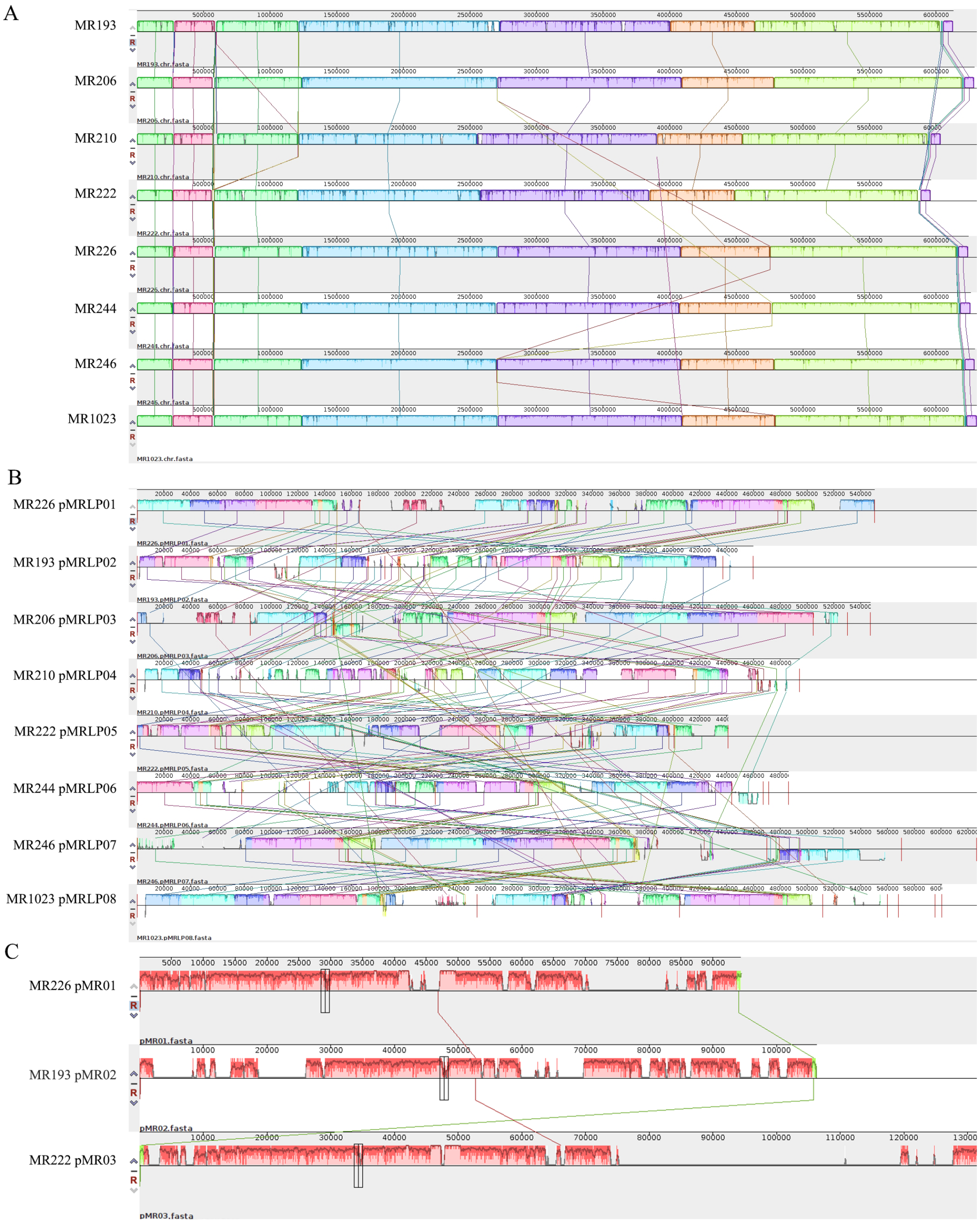

Figure S3: Multiple alignment of 8 *M. riyadhense* assemblies using progressive Mauve (1)**.**

Each *M. riyadhense* genome is laid out horizontally with homologous fragments (locally collinear blocks, LCBs) outlined as colored rectangles. (A) The alignment of the chrmosomes from 8 *M. riyadhense* strains,

(B) the alignment of the linear plasmids (pMRLP01-08) from 8 strains. (C) the alignment of the circular plasmids (pMR01-03).

A

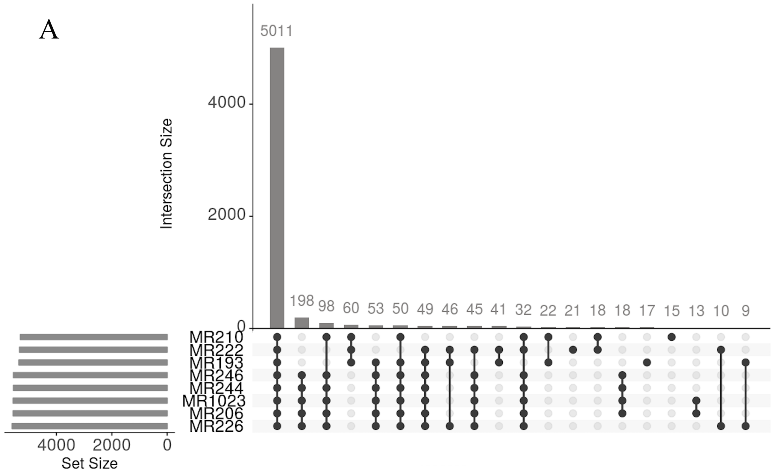

B

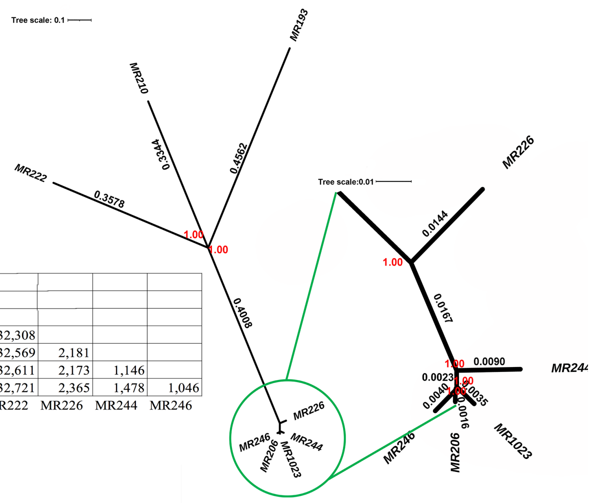

	MR193	MR206	MR210	MR222	MR226	MR244	MR246
MR206	32,696						
MR210	32,955	28,885					
MR222	33,454	32,582	29,699				
MR226	32,192	2,198	28,507	32,308			
MR244	32,439	1,249	29,034	32,569	2,181		
MR246	32,683	667	28,986	32,611	2,173	1,146	
MR1023	32,827	899	29,056	32,721	2,365	1,478	1,046

C

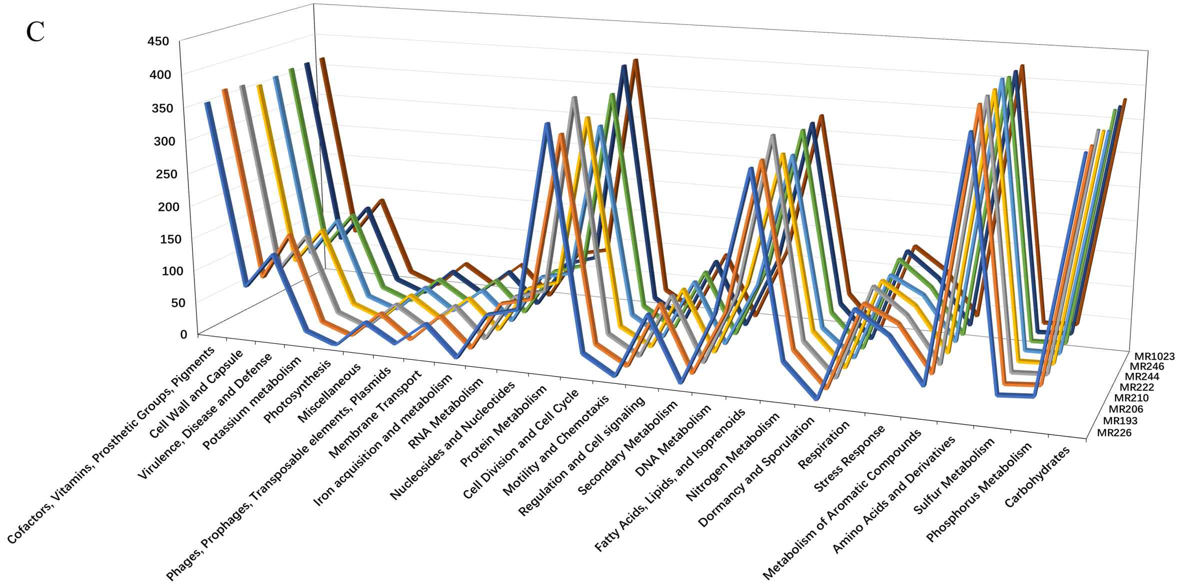

Figure S4: Phylogenetic tree and genome content comparison of *M. riyadhense* clinical isolates used in this study.

(A) UpSet Venn diagrams of the top 20 clusters of orthologous/paralogous groups identified across the 8 *M. riyadhense* genomes. (B) Phylogenetic tree of *M. riyadhense* clinical isolates used in this study. Part of the phylogeny (green circle) was enlarged for visualization purpose. The branch length are labled in black on each branch and the boostrap values are labeled in red for each node. The *M. riyadhense* phylogenetic tree was constructed with SNPs data from 8 datasets called by GATK pipeline (2) by RaxML with the TVM model(3). The circles on each branch indicate the bootstrap values. The pairwise SNPs counts are recorded in the table. (C) The distribution of the subsystem-features annotated by SEED viewer (4)of the predicted protein-encoding genes across 8 *M. riyadhense* strains.

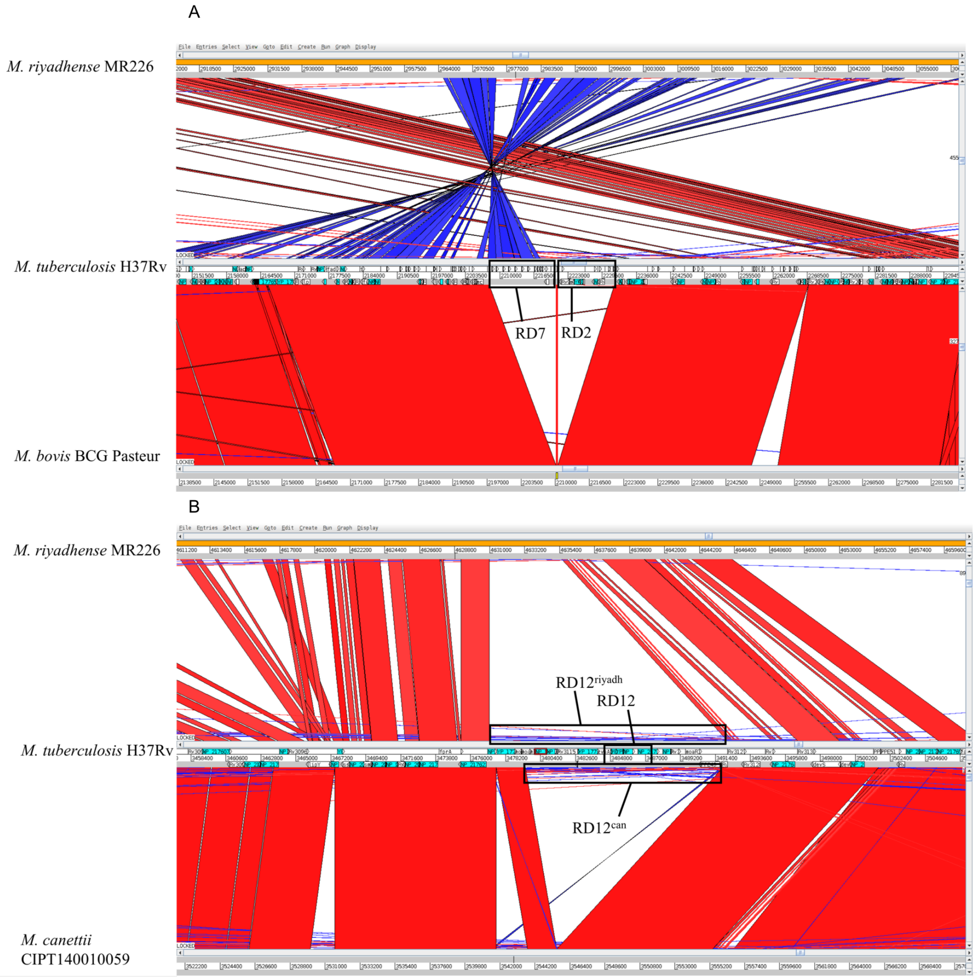

Figure S5: Genome alignments comparing selected Region of Differences (RDs) in mycobacteria. The Artemis Comparison Tool (ACT) (5)was used to compare and visualize the annotated genome sequences against the chosen mycobacteria.

(A) The RD2 region of *M. riyadhense* MR226, *M. tuberculosis* H37Rv and *M. bovis* BCG Pasteur (B) The RD12 region of *M. tuberculosis*, *M. riyadhense* MR226 and *M. canettii* CIPT 140010059.

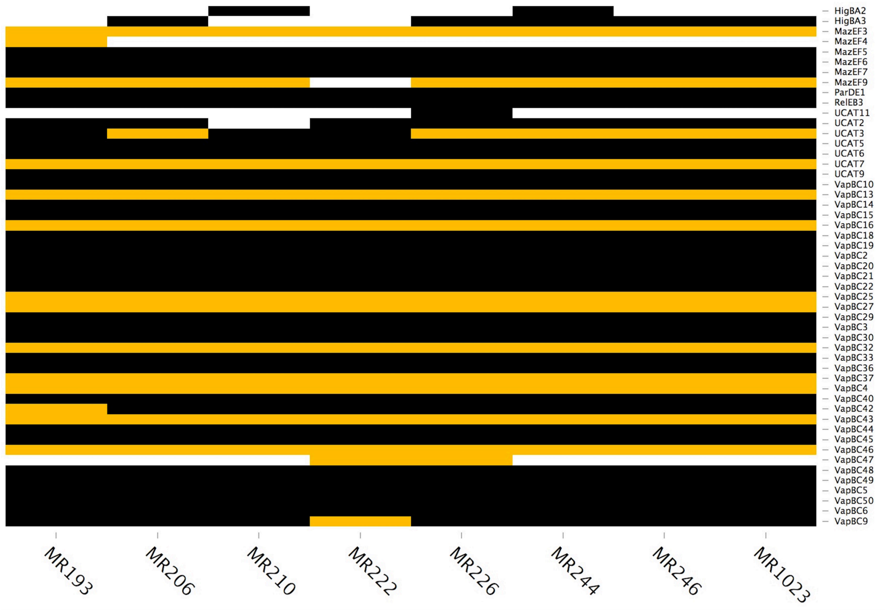

Figure S6: Presence (black) and absence (white) of *M. tuberculosis* H37Rv toxin/antitoxin orthologs in eight sequenced *M. riyadhense* clinical strains. The orange blocks denote the presence of either the toxin or antitoxin orthologue in a given pair of the T/A system. The black and white blocks represent presence and absence respectively. The name of the T/A system are shown for each row on the right.

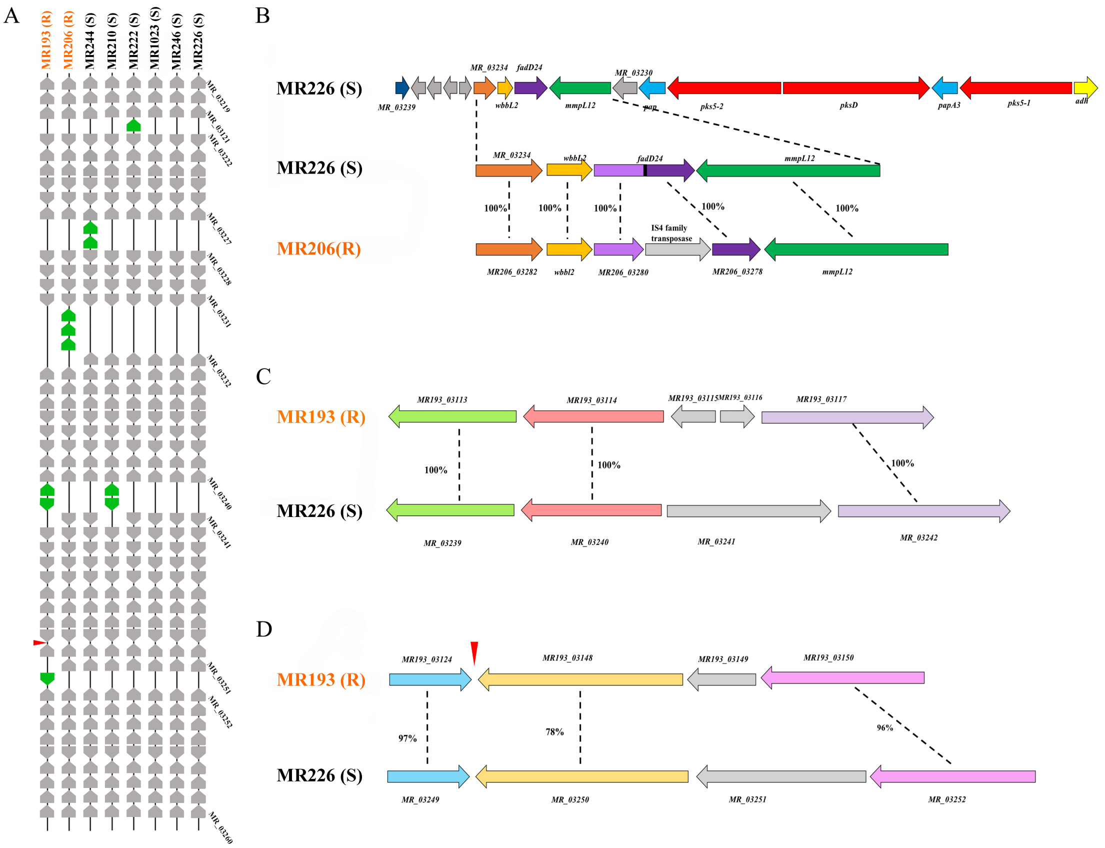

Figure S7: The locus map of the LOS biosynthesis gene cluster in 8 sequenced *M. riyadhense* strains showing variations in the rough strains.

The rough or smooth strains are donoted by '(R)' and '(S)' letters respectively after the strain name in parentheses. The rough stains are highlighted with orange labels. (A) Genetic locus map of the LOS biosynthesis gene cluster of 8 *M. riyadhense* strains. The genes are shown with arrows. The green arrows indicates presence of variation i.e. presence/absence of CDS for each strain. (B) Part of the LOS biosynthesis gene cluster of MR206 strain. The insertion sequence is zoomed in for illustrative purposes. An IS4 family transposase insertion has been detected in the *fadD24* gene of the MR206 LOS biosynthesis gene cluster. (C) Part of the LOS biosynthesis gene cluster of MR193 strain. The ortholog of *MR_03241*, a glycosyl transferase is missing in the rough strain MR193 and also in one smooth strain MR210 (shown in (A)). (D) Part of the LOS biosynthesis gene cluster of MR193 strain. A 23 gene insertion fragment (several transposases, integrases and IS elements have been detected in this region) has been found in MR193 between *MR193_03124* to *MR193_03148*. *MR_03251* is missing in the LOS locus of MR193. *MR193_03251* is annotated as a glycotransferase that doesn't have any orthologues in *M. canettii*, *M. tuberculosis* or *M.*

marinum. Homologous regions between the cloned sequences in (B)(C)(D) are in the same color (except for the grey color, which represents the gene that doesn't have any orthologues within the LOS locus) and linked by a dashed line and the similarity scores are shown.

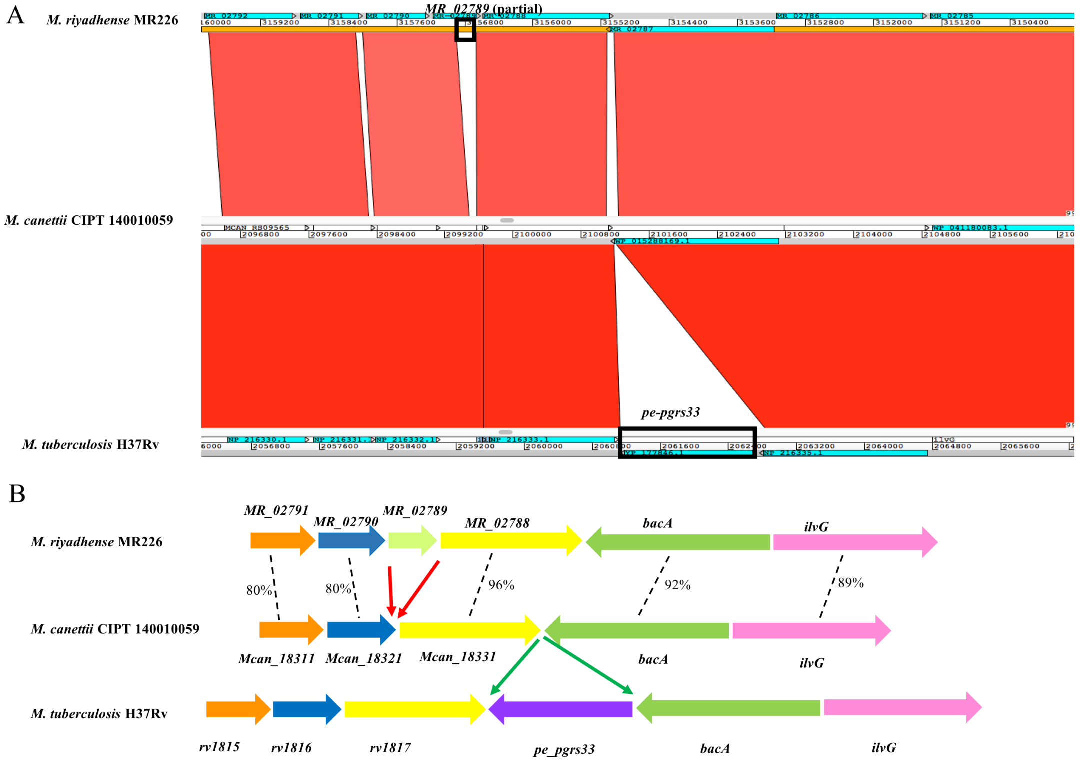

Figure S8: Comparison of the gene content of *M. riyadhense* strain MR226, *M. canettii* strain CIPT 140010059 and *M. tuberculosis* H37Rv in the *pe-pgrs33* region.

(A) Genome alignments comparing *pe-pgrs33* region in mycobacteria. The Artemis Comparison Tool (ACT) was used to compare and visualize the annotated genome sequences against the chosen mycobacteria. (B) Genetic locus map of the *pe-pgrs33* gene cluster from *M. riyadhense*, *M. canettii* and *M. tuberculosis* (drawn to scale). Homologous regions between the cloned sequences are linked by a dashed line and the similarity scores are shown. The genes are shown with arrows and are colored according to the orthologs. The deletion event was highlighted with red arrows while the insertion event with green arrows.

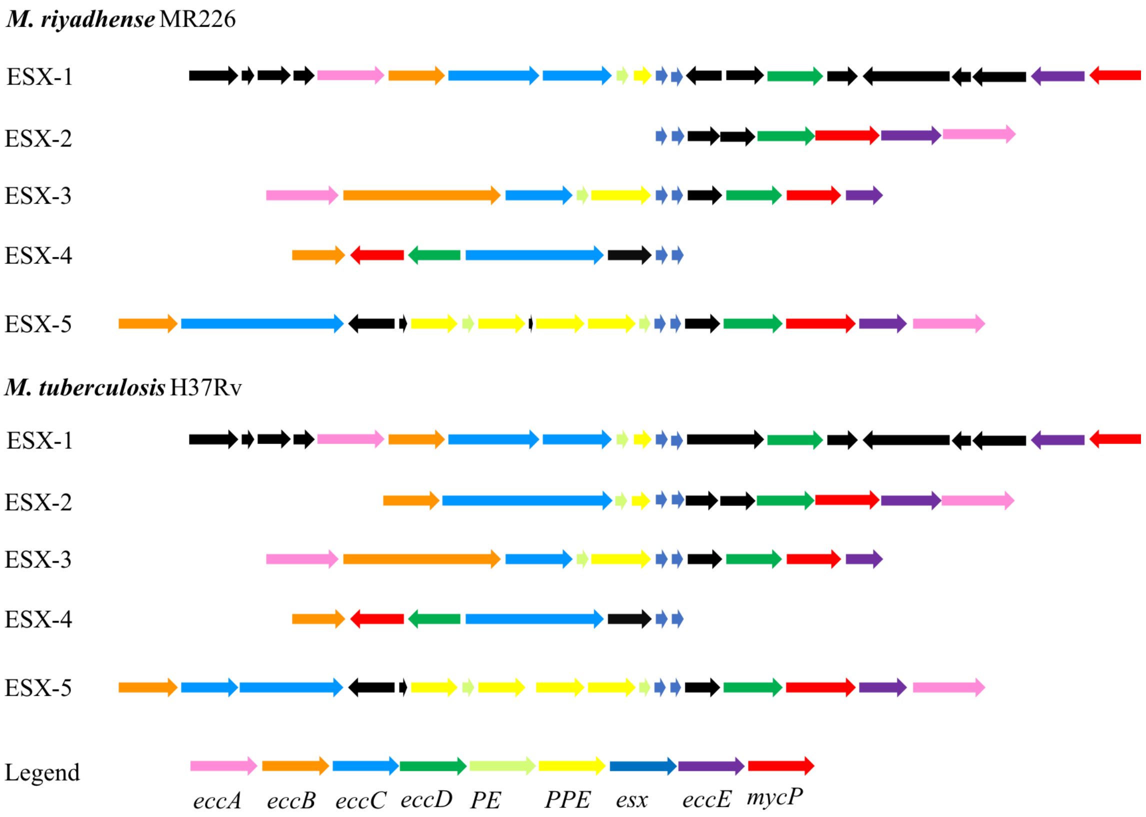

Figure S9: Comparison of different gene clusters that encode type VII secretion systems in the *M. riyadhense* MR226 and *M. tuberculosis* H37Rv.

The genes are shown with arrows and are colored according to the orthologs. The color codes for the figure are presented in the key. The black arrows indicate region-specific genes.

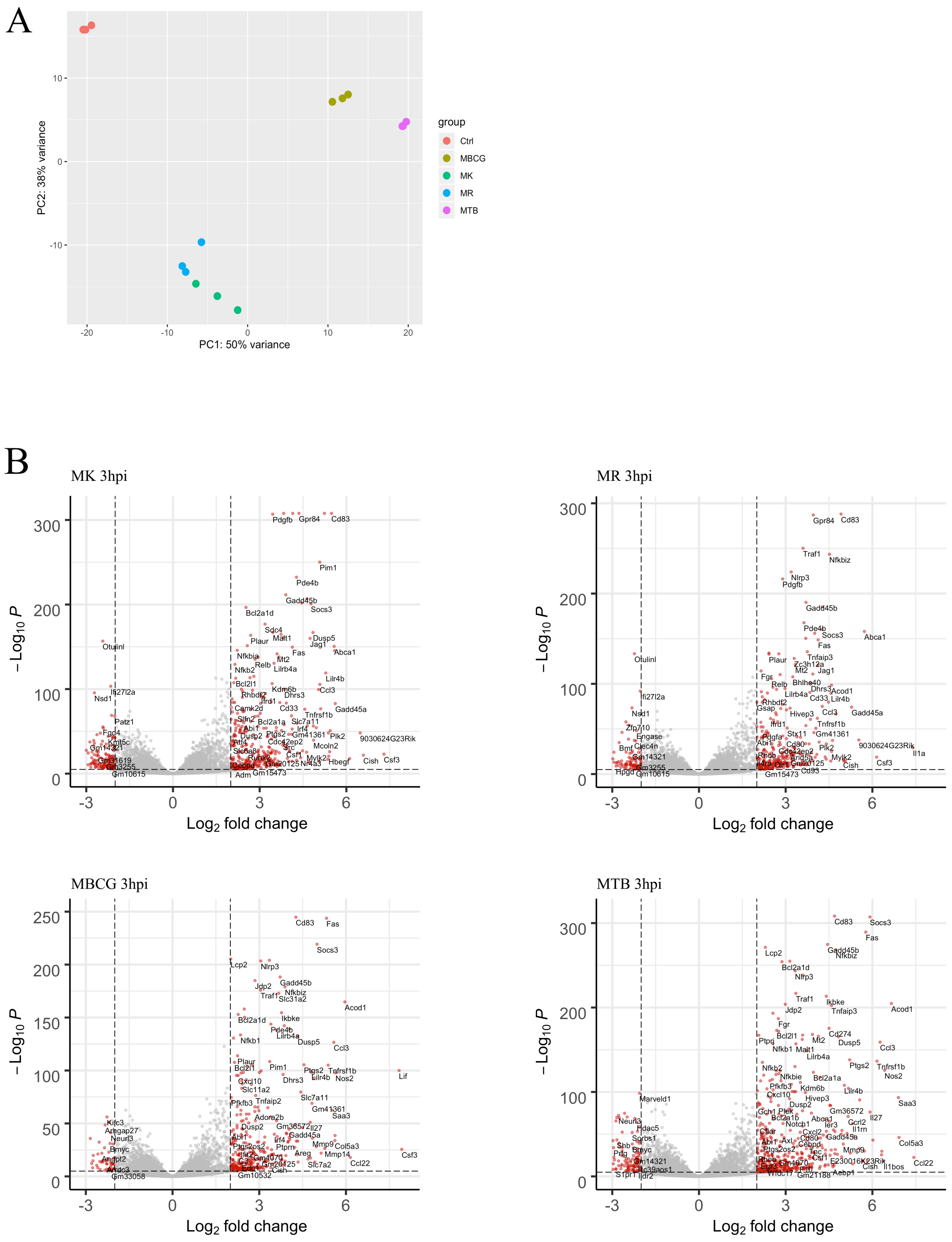

Figure S10: Transcriptome responses of RAW264.7 cells at 3 hours post infection.

(A) Principal components analysis based on the transcriptome data of RAW264.7 cells infected with *M. bovis* BCG (MBCG), *M. kansasii* (MK), *M. riyadhense* (MR) *M. tuberculosis* (MTB), or non-infected control samples (Ctrl) at 3 hpi. (B) Corresponding volcano plots (p value versus fold change ratio bacteria/control). Red dots are significant at $p = 0.01$. A selection of genes, both up-and down-regulated, are identified as shown.

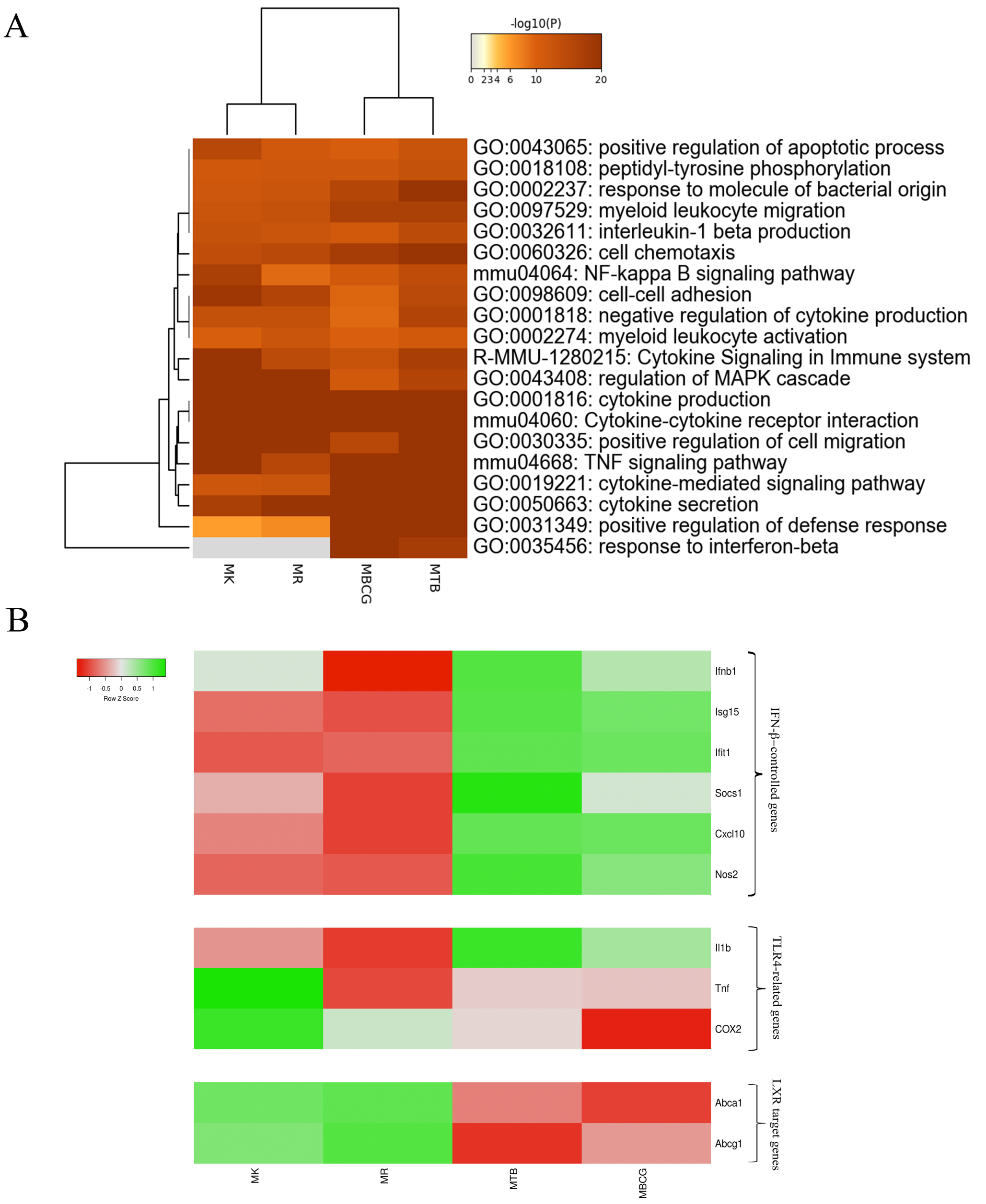

Figure S11: Comprehensive pathway analysis of RAW264.7 cells upon infections.

(A) Heatmap of top 20 significantly enriched GO terms of host cell response across different bacterial infections, colored by p-values at 3hpi. (B) Heatmap of RNA-Seq expression z-scores computed for IFN-β-controlled genes (upper panel), and genes related to TLR4 (middle panel) or Liver X receptors (lower panel) in RAW264.7 cells; comparing each of the 4 mycobacterial infections with non-infected controls 3 hpi.

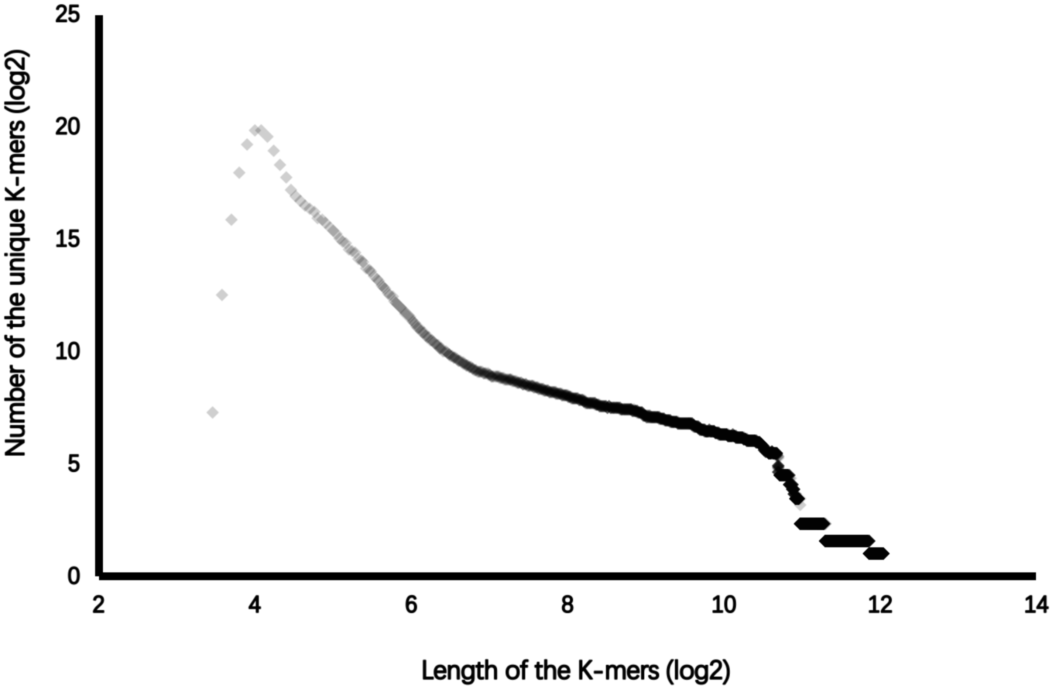

Figure S12: Basic statistics of Unique K-mers of *M. riyadhense* across 152 mycobacteria genome assemblies.

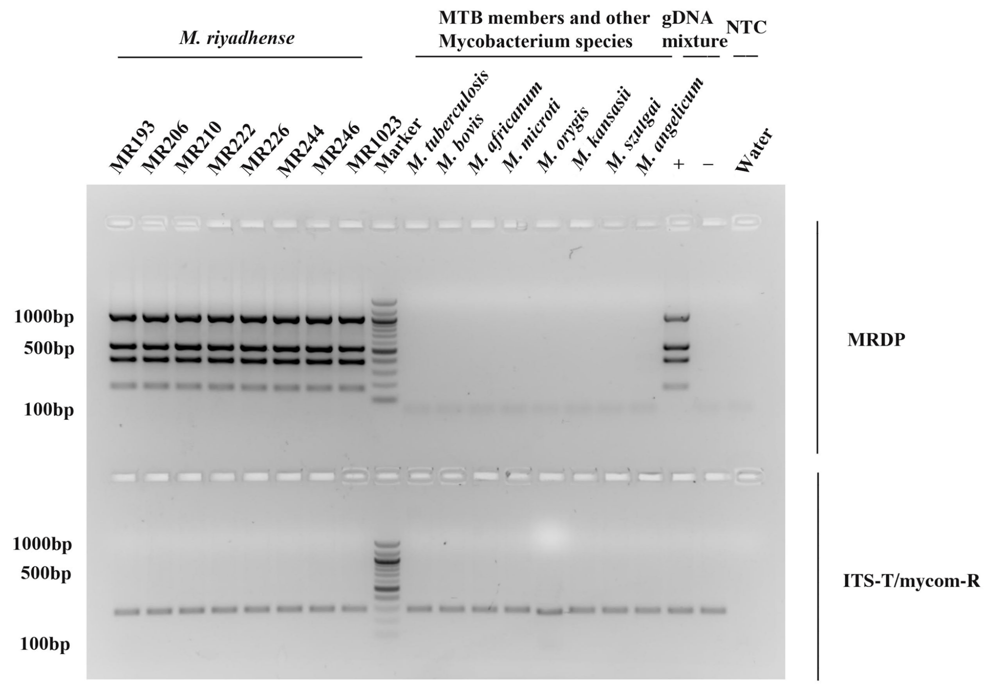

Figure S13. Development of a rapid PCR-based diagnostic test for detection of *M. riyadhense*.

Agarose gel (2%) electrophoresis patterns of the PCR products are shown as part of the diagnostic test for *M. riyadhense*. Lane M: DNA Marker, Lane 1-8: Varies *M. riyadhense* strains (From left to right: MR193, MR206, MR210, MR222, MR226, MR244, MR246, MR1023), Lane 9-16: Varies *Mycobacterium* species (From left to right: *M. tuberculosis*, *M. bovis*, *M. africanum*, *M. microti*, *M. oryis*, *M. kansasii*, *M. szulgai* and *M. angelicum*)

templates, Template cocktail, mycobacterium species (*M. tuberculosis*, *M. bovis*, *M. kansasii*, *M. marinum*, *M. szulgai*, *M. avium* and *M. angelicum*) with (Lane 17, +) and without (Lane 18,-) *M. riyadhense* MR226 gDNA template. Lane 19: Non-template control (NTC). Upper Panel: MRDP (*M. riyadhense* diagnostic marker) set. Lower Panel B: *Mycobacterium* genus specific primer ITS-T and mycom-R amplified with mycobacterial gDNA.

REFERENCES:

1. Darling,A.C.E., Mau,B., Blattner,F.R. and Perna,N.T. (2004) Mauve: Multiple alignment of conserved genomic sequence with rearrangements. *Genome Res.*, **14**, 1394–1403.

2. Alkan,C., Coe,B.P. and Eichler,E.E. (2011) GATK toolkit. *Nat. Rev. Genet.*, **12**, 363–76.

3. Stamatakis,A. (2014) RAxML version 8: A tool for phylogenetic analysis and post-analysis of large phylogenies. *Bioinformatics*, **30**, 1312–1313.

4. Overbeek,R., Begley,T., Butler,R.M., Choudhuri,J. V., Chuang,H.Y., Cohoon,M., de Crécy-Lagard,V., Diaz,N., Disz,T., Edwards,R., *et al.* (2005) The subsystems

approach to genome annotation and its use in the project to annotate 1000 genomes. *Nucleic Acids Res.*, 10.1093/nar/gki866.

5. Carver,T., Berriman,M., Tivey,A., Patel,C., Böhme,U., Barrell,B.G., Parkhill,J. and Rajandream,M.A. (2008) Artemis and ACT: Viewing, annotating and comparing sequences stored in a relational database. *Bioinformatics*, 10.1093/bioinformatics/btn529.
